# Interpreting Microbial Biosynthesis in the Genomic Age: Biological and Practical Considerations

**DOI:** 10.3390/md15060165

**Published:** 2017-06-06

**Authors:** Ian J. Miller, Marc G. Chevrette, Jason C. Kwan

**Affiliations:** 1 Pharmaceutical Sciences Division, School of Pharmacy, University of Wisconsin-Madison, Madison, WI 53705, USA; ijmiller2@wisc.edu; 2 Department of Genetics, Department of Bacteriology, University of Wisconsin-Madison, Madison, WI 53706, USA; chevrette@wisc.edu

**Keywords:** genome mining, genome sequencing, metagenomics, binning, biosynthesis, bioinformatics, biosynthetic gene clusters, secondary metabolism

## Abstract

Genome mining has become an increasingly powerful, scalable, and economically accessible tool for the study of natural product biosynthesis and drug discovery. However, there remain important biological and practical problems that can complicate or obscure biosynthetic analysis in genomic and metagenomic sequencing projects. Here, we focus on limitations of available technology as well as computational and experimental strategies to overcome them. We review the unique challenges and approaches in the study of symbiotic and uncultured systems, as well as those associated with biosynthetic gene cluster (BGC) assembly and product prediction. Finally, to explore sequencing parameters that affect the recovery and contiguity of large and repetitive BGCs assembled *de novo*, we simulate Illumina and PacBio sequencing of the *Salinispora tropica* genome focusing on assembly of the salinilactam (*slm*) BGC.

## 1. Introduction

As sequencing costs continue to decrease [1], it is now more feasible than ever to sequence the genome of natural product producing organisms. For isolated strains, the use of long-read PacBio sequencing combined with short-read Illumina data is now the gold standard, frequently yielding completely assembled microbial genomes using off the shelf assemblers [1,2,3]. Such technology provides access to genomic information that can be readily mined for new biosynthetic pathways, be they active or silent. However, there are situations when sequencing and assembly are not as readily accomplished. For instance, it may be difficult to extract large enough quantities of high-quality DNA from some systems (e.g., the variable cellular rigidities and doubling times of many Actinobacteria [4]); this limitation particularly impacts applications of culture-independent sequencing (metagenomics). In this review, we outline biological and practical issues to consider when embarking on a sequencing project to yield small molecule biosynthetic pathways. We also investigate the factors that contribute to successful assembly of repeat-laden biosynthetic pathways.

Natural product chemists often desire to sequence biosynthetic pathways for a number of interconnected reasons. The most basic motivation is perhaps the gleaning of structural information from sequence data. In particular, absolute stereo-configuration can be predicted from the sequence of modular pathways such as polyketide synthase (PKS) and nonribosomal peptide synthetase (NRPS) systems [5]. Such analyses can be used to assign probable configurations when they are recalcitrant to spectroscopic and chemical analyses; this is especially the case with polyketides. The genomic context of a pathway may also give clues as to the molecular target or mechanism of action of a compound, since genes involved in resistance mechanisms are often clustered with natural product biosynthetic genes [6,7]. Another motivation for sequencing pathways is to establish a renewable supply of the compound of interest, either through engineering of the producing organism [8,9], or by heterologous expression [10]. Depending on the structural complexity of the natural product and/or the biosynthetic machinery driving its synthesis, this approach to production may be more practical than total organic synthesis.

Notably, shotgun (random) sequencing campaigns generally associated with cluster identification often unearth much more data besides the sequence of a single pathway. These data can include the entire genome of the producing organism. In the case of metagenomics projects, the genomes of other co-localized species often complicate or obscure the specific pathway of interest. Nevertheless, this information can tie primary [11] and secondary metabolic pathways [12,13] to a specific organism allowing one to investigate the producing organism’s ecology and/or evolutionary history [14]. For instance, the degree of genome reduction in microbial symbionts [15,16,17] can suggest approximate evolutionary age and dependency of the symbiosis, along with the natural products made by the symbiont. One can also carry out comparative studies to investigate the function and evolution of natural products in the environment [18], distribution of pathways through horizontal transfer [19], and the dynamics of pathway expression in the environment [20,21]. Sequencing, therefore, can be used to study many aspects of chemical ecology, which is often of great interest to natural product chemists since the evolved target may be related to therapeutically relevant activities [22].

For these and other reasons, there is currently a great deal of interest in the application of “omics” technologies in the natural products field. Rather than exhaustively covering all “omics” work related to natural products, we concentrate herein on the limitations of current methods and the caveats in data analysis that researchers embarking on sequencing projects need to be aware of when designing experiments and analyzing acquired sequence data. We also discuss some biological, evolutionary, and ecological factors warranting consideration throughout the course of sequencing projects.

## 2. Evolution of Biosynthetic Pathways

The genes driving microbial secondary metabolism are typically, but not always, clustered; genes involved in the biosynthesis, modification, transport, and regulation of a particular metabolite are generally adjacent to one another on the chromosome. These biosynthetic gene clusters (BGCs) are often complex and can be larger than 100 kilobases with numerous operons under tight regulatory control. The evolutionary mechanisms that drive gene clustering observed within BGCs remain unclear. One prevailing hypothesis suggests that genes conveying a fitness benefit (e.g., via the production of an antibiotic natural product) will tend to cluster over evolutionary timeframes due to the importance of their “teamwork” [23] in generating a compound that endows a benefit upon the producing species and/or any of its symbiotic partners. Biosynthetic potential is a function of the environmental chemical landscape [24] and species–species interactions [22,25] that define an organism’s niche. However, the spatial and temporal dynamics of microbial interaction networks and selective forces are largely unknown and rational discovery strategies that leverage ecological interactions have only begun to be employed in a few relatively well-defined systems [4,25,26,27].

BGCs are widely distributed among microbes [4,19,28,29] and approximately 7% of bacteria dedicate 7.5% or more of their genomes to secondary metabolism [30]. Over 6000 broad BGC families have been described and their discontinuous presence-absence patterns suggest that gains and losses occur frequently over evolutionary timescales [30]. BGCs also exhibit high rates of insertions, deletions, duplications and rearrangements [19], often exchanging multi-gene blocks with primary metabolism [31] or other BGCs [19]. Shared loci within functional domains, many of which contribute to metabolite chemistry, are under a wide array of selective pressures both across and within clusters [32].

The modular biosynthetic logic, high GC content, and high extents of repetition within polyketide synthase (PKS) and nonribosomal peptide synthetase (NRPS) BGCs further results in distinct module and domain level exchanges that influence metabolite chemistry [19,28,33,34,35,36]. In the type 1 PKS avermectin cluster from *Streptomyces* sp., BGC variations from strain to strain include functional domain exchanges (dehydratase-ketoreductase units) as well as losses and gains (ketoreductases) [36]. These rearrangements are thought to stem from homologous sequences within interdomain linkers [36]. Across type 1 PKSs, the sequences of ketosynthase domains tend to group phylogenetically based on the BGC in which they are contained [36] suggesting that intracluster duplication of domains is an important mechanism of type 1 PKS evolution. In contrast, the phylogeny of the ketosynthase domains from *trans*-acting acyltransferase PKSs tends to group on the basis of accepted substrate structures [37], suggesting that lateral gene transfers are important in the evolution of domain chemistry within these BGCs. Subtle mutations within adenylation domains have led to substrate shifts within evolutionarily-related NRPS BGCs [27]. Notable in this regard is the isoleucine to valine shift between massetolide, orfamide, and viscosin (Ile-9, Val-10, and Ile-9, respectively) described recently in *Pseudomonas* [38]. Similarly, nonsynonymous point mutations in the precursor peptide regions of ribosomally synthesized and post-translationally modified peptides (RiPPs) can have significant structural consequences within the final product [39] that can impact the producing organism’s fitness. As a result of high substrate promiscuity within the supporting biosynthetic machinery, RiPP families, such as the cyanobactins, can exist in nature as “combinatorial libraries”, where hypervariable precursor peptide genes are deployed across the fitness landscape, while relaxed-specificity tailoring enzymes are conserved [40]. Although the vast majority of microbial biosynthesis remains to be described [28,41], work to date suggests that the diverse mechanisms underlying BGC evolution are both cluster- and context-dependent.

## 3. Pathways from Symbiotic and Uncultured Sources

It has long been known that culture-based studies vastly underestimate microbial diversity in the environment, a phenomenon known as the “great plate anomaly” [42]. The exact fraction of microbial biodiversity amenable to laboratory culture is still being debated [43], especially in light of efforts using novel culturing techniques [44,45,46]. However, from culture-independent sequencing efforts, it is estimated that there are over 1000 bacterial phyla [47,48], and only a small fraction have ever been cultured. We cannot know how much microbial biodiversity remains undiscovered, but much of this “microbial dark matter” [49] is likely to only be detectable through culture-independent sequencing with so called “meta-omics” [50] techniques, including metagenomics and metatranscriptomics. Our view of the extent of “microbial dark matter” was first shaped by amplicon studies where 16S ribosomal RNA genes were amplified directly from the environment [51]. Used as a phylogenetic marker, 16S amplicon sequences give a measure of bacterial biodiversity and allow the species composition of different environments to be compared. However, large metabolic differences can be seen between strains with near identical 16S sequences [52,53], highlighting that 16S amplicon-based studies lack the genomic resolution necessary to elucidate the ecology and lifestyle of microbes in their natural systems. Additionally, it has recently been shown that a significant portion of the bacterial tree of life is inaccessible to standard 16S primers [48].

Shotgun (random) sequencing technology has now progressed to the point where whole genomes of uncultured bacteria can be extracted from complex metagenomes [48,54]. In nature, microbes do not generally live as monocultures and mixed communities can be quite complex, leading to several challenges in meta-omics. Because such communities can contain many microbial genomes (and perhaps the genome of a eukaryotic host), high sequencing depth is required in order to obtain adequate read coverage for individual genomes. Assembling large sequencing datasets can be demanding of computational hardware and assembly algorithms, which generally scale to the number of unique “*k*-mers” (where *k* is the sequence length) in the dataset [55,56,57,58]. This phenomenon is especially true for complex metagenomes [58]. Finally, after assembly is achieved, deconvolution of larger assembled genome fragments, known as “contigs”, into discrete genomes (often referred to as “bins”) remains a challenging bioinformatics problem (see below).

The biosynthetic diversity of uncultured “microbial dark matter” has been explored using two major culture-independent approaches. The first approach entails random functional screens of metagenomic clone libraries to find novel heterologously expressed natural products [59,60]. The second approach involves targeted sequencing of systems known to produce interesting natural products [50,61,62,63,64]. There are relatively few examples of natural product discovery solely through sequencing of a pathway from an uncultured microbe. Notably, diaphorin [65] and nosperin [66] were characterized after their respective pathways were uncovered and found to be related to those of pederin [67,68], psymberin [69] and onnamide A [70]. A number of natural products have also been identified through genome mining of the human microbiome [71,72]. Recently, the Brady group has synthesized 288 peptide structures predicted from NRPS pathways found in publicly available bacterial genomes; several were active against ESKAPE pathogens [73]. As the fields of metagenomics, bioinformatics, and synthetic biology [74,75] continue to advance, this sequence-driven route of natural product discovery is likely to become more prominent. 

Random functional screens (i.e., “functional metagenomics” [76]) generally focus on environments such as soil that contain very complex microbial communities. Recent findings suggest that this complexity is mediated by opposing forces of production, resistance, and degradation of the diverse antibiotics produced by soil microorganisms [77,78]. Functional screens attempt to capitalize on this biosynthetic diversity by cloning and expressing genes taken directly from environmental samples. In a functional screen, DNA is extracted from an environmental sample and a library of clones is made and transformed into some sort of heterologous host such as *Escherichia coli* [79], *Streptomyces lividans* [80], or fungal expression systems [81]. Transformed colonies are screened for the effects of expressed compounds, such as pigmentation of colonies [76] or antibiotic effects on a target organism [82]. There are a number of limitations to this approach: pathways must be smaller than the clone insert size and clustered into a discrete chromosomal region, they must be functionally expressed in the heterologous host, and their products must not kill this host. Nevertheless, functional metagenomics screening has yielded small molecules, such as the terragines [80], antibiotic long-chain *N*-acyl amino acids [82], and commendamide [83]. 

Targeted sequencing efforts tend to focus on systems where microbes live in a symbiotic relationship with a eukaryotic host. This interest is fueled both by the many known natural product isolations from eukaryotes and by the fact that these hosts can harbor stable symbiotic communities, the genomes and secondary metabolites of which can be more reproducibly obtained, compared to those of their free-living microbial counterparts [84]. Microbial communities not associated with a higher organism pose a problem for recollection, except perhaps for certain lichens [66] and cyanobacterial assemblages [85]. Although the focus on symbiotic microbes is, on some level, practical, there is an ecological rationale to study small molecules made by symbionts. Very often compounds isolated from these systems have bioactivities suggestive of a defensive function, such as cytotoxicity, that has presumably been honed through millions of years of evolution and selective pressures. This phenomenon implies that for a symbiotic relationship to be established and maintained across evolutionary timescales, the natural products produced by symbionts must have ecologically important bioactivities. Following this line of thought, the level of “importance” of a natural product could be suggested by determining the interdependence of the symbiotic partners. 

There is a continuous spectrum of dependency on both sides of a symbiotic relationship, roughly proportional to evolutionary timeframe as well as exclusivity. For instance, the relationship between eukaryotic cells and mitochondria, believed to be ~1.2 billion years old [86], is completely exclusive, and is essential to both parties. On the other end of the scale, symbiotic relationships can be more transient or the degree of dependency can be unequal (commensal or even parasitic) for the different partners. For example, arbuscular mycorrhizal (AM) fungi associate with plant roots, facilitating water and nutrient uptake [87]. Although AM fungi are dependent on plants for growth, the presence of AM fungi is beneficial but not essential for plant growth, and there are whole plant lineages that appear to have diverged from this kind of symbiosis. Co-evolution of symbiont and host can lead to a state where the symbiont is exclusive to and dependent on the host [15,16]. This process is driven by the loss of genes that are not required for life outside the host. Such a scenario precludes independent life or culture in the laboratory and implies that any biosynthetic pathway maintained throughout the process of genome reduction and gene loss, is under strong selective pressure and has been ecologically important across evolutionary timescales. Ecologically important natural products from long-term symbionts, therefore, are likely to have evolved specific biological activities that may also be useful in therapeutic settings [22,88]. Many of the examples outlined below have cytotoxic activities suggesting defensive roles in the environment as well as anti-cancer drug potential.

Although biosynthetic pathways in free-living bacteria are generally clustered, there are some notable exceptions. An early example of a non-clustered pathway came with the discovery of two discrete gene clusters that are both required for the biosynthesis of ansamitocin in *Actinosynnema pretiosum* [89]. Similar fragmentation has been reported in a number of symbiotic systems. For example, the biosynthetic pathway for the defensive compound pederin is split into at least two loci [67] in the genome of a *Pseudomonas* sp. symbiont of blister beetles. Intriguingly, related compounds and pathways have been found in other insects, marine sponges, and a lichen [37], suggesting that these pathways were originally acquired horizontally. The presence of decayed insertion sequences flanking the pederin biosynthetic loci suggest that the fragmentation of the pathway resulted from genome rearrangements following horizontal acquisition [90]. Another pederin variant, diaphorin, is biosynthesized by a bacterial symbiont of a psyllid pest of citrus crops, *Diaphorina citri* [65]. The symbiont, “*Candidatus* Profftella armatura”, has a highly reduced genome less than 500 kbp in size, yet the diaphorin pathway occupies 15% of the genome in two loci [65]. Intracellular symbionts, such as “*Ca.* P. armatura”, are especially prone to extensive genome reduction, which eventually leads to an inability to rearrange their genome or accept horizontally transferred genes. A similarly-reduced intracellular symbiont, *Buchnera aphidicola*, has been associated with aphids for an estimated 160–280 million years [91], and for the past 50–70 million years no rearrangements or gene acquisitions have occurred [92]. Therefore, the diaphorin pathway was likely acquired horizontally early in the evolution of the symbiotic relationship between “*Ca.* P. armatura” and *D. citri*. 

There are a number of other examples of pathway fragmentation in symbionts. The bryostatins are cytotoxic polyketides known to protect the vulnerable larvae of the bryozoan *Bugula neritina* from predation [93], and these compounds are made by a bacterial symbiont, “*Candidatus* Endobugula sertula”. The bacterium is disseminated vertically with released larvae, and there are a number of genetically isolated populations of *B. neritina* that harbor distinct genotypes of “*Ca.* E. setula” [93,94,95]. The bryostatin BGC (*bry*) was sequenced through clone library methods by the Haygood and Sherman groups [96,97]. Interestingly, the described *bry* BGC exists as a continuous locus in the “shallow” sibling species of *B. neritina*, but is fragmented into two loci in the “deep” genotype. More recently, the entire genome of “*Ca.* E. sertula” was sequenced and two additional *bry* genes were found in a distal locus in the “shallow” genotype [17]. Likewise, a core locus of the ET-743 pathway was determined through shotgun sequencing [50], but the genome of the bacterial symbiont “*Candidatus* Endoecteinascidia frumentensis” had to be completed in order to identify all genes in the pathway, found across multiple loci [62]. A similar level of fragmentation is observed in the patellazoles pathway, found in the intracellular tunicate symbiont “*Candidatus* Endolissoclinum faulkneri”, where *ptz* genes are distributed between seven distinct loci in the genome [63]. Fragmentation has also been found in a terrestrial fungus-growing ant system, where several bacterial symbionts produce related compounds dentigerumycin and the gerumycins, some of which have significant antifungal activity against the microfungal pathogen *Escovopsis* sp.; this activity spares the fungal crop grown for food by the ants [98]. The pathways for dentigerumycin and the gerumycins have apparently been acquired recently through horizontal transfer. However, in one strain of *Pseudonocardia* sp., the gerumycin pathway is split into two loci on a plasmid, whereas this pathway occurs as a contiguous cluster in another strain. The related dentigerumycin cluster is also a contiguous cluster in a third strain. Thus, the fragmentation may have occurred recently. Alternatively, the fragmented version may represent the origin of the contiguous pathway from simpler components.

The above examples underscore the importance of both metagenomic assembly and binning when attempting to extract biosynthetic pathways from symbiotic systems. Because biosynthetic pathways in symbiont genomes tend to be fragmented, it is important to accurately identify all contigs belonging to the symbiont genome of interest. The challenges of assembling and binning may also vary with the age and/or host-restriction of the symbiosis. Symbionts with reduced genomes that have been vertically transmitted and host-restricted for millions of years tend to accumulate mutations due to frequent population bottlenecks, weak purifying selection and eventual loss of DNA repair mechanisms [16]. Consequently, previously repetitious regions tend to diverge. For example, the complete chromosome of two strains of the patellazole-producing symbiont “*Ca.* E. faulkneri” was assembled twice relatively easily and independently from two complex tunicate metagenomes [18,63]. Both of these strains have low coding density, with intergenic regions showing significantly different GC content than protein and RNA coding genes. The distinct intergenic sequence composition suggested by different GC content and lack of DNA repair pathways with consequent sequence drift likely led to unique and unambiguous *k*-mer paths (de Bruijn graphs [55]), allowing the assembler to yield more contiguous and near complete genome assemblies, free of interference from other species. In contrast, the bryostatin-producing symbiont “*Candidatus* Endobugula sertula” is likely to be less host-restricted because its genome shows few signs of genome reduction [17], and horizontal transfer may be possible in addition to the vertical mode [99]. Consequently, the symbiont’s genome was more of a challenge to assemble. The *bry* cluster exhibits a more challenging repeat structure, because its functional DNA repair pathways and perhaps the relatively short time since *bry* acquisition have prevented these repeats from significantly diverging [17,97]. One additional consideration is that, as symbiont genomes contract, annotation of fragmented biosynthetic pathways can become less challenging and less ambiguous. For instance, when there is clearly only one surviving secondary metabolite pathway and few primary metabolic pathways present, it is easier to determine which distal genes are likely to work together to make a particular natural product, even if they are fragmented across the genome.

## 4. Challenges in Biosynthetic Pathway Assembly and Product Prediction

### 4.1. Capabilities and Limitations of Current Sequencing Technologies

There are a number of sequencing technologies currently available, generally characterized by tradeoffs involving possible read lengths and coverage depths. These technologies have been extensively reviewed elsewhere [1,100]. Accordingly, we will only cover selected practical considerations herein. The current standard short-read (~50–250 bp), high-coverage technique used for both genomics and metagenomics is Illumina sequencing. Due to the PCR amplification step within Illumina workflows, and the ease of generating small-insert libraries from low amounts of input material, Illumina is well-suited for shotgun metagenomic and metatranscriptomic sequencing. It is challenging to extract large amounts of DNA from metagenomic samples; often such efforts yield highly sheared molecules. The primary disadvantages of short-insert, short-read data is that the connectivity across repetitive regions is challenging, if not impossible, to resolve. Long-read technologies (such as PacBio and Oxford Nanopore) are generally single-molecule techniques, meaning that PCR amplification is not employed during sequencing. Thus, these techniques generally require much higher DNA input amounts (often up to tens of micrograms) and higher quality (high molecular weight fragments) as only a subset of extracted DNA is sufficiently long to take advantage of reads ≥50 kbp in length. Another distinct disadvantage of long-read technologies is the lack of coverage. Whereas Illumina is capable of producing ~200 M paired-end reads from a single lane, PacBio Sequel instruments can yield ~1 M reads from each Single Molecule Real Time (SMRT) cell. Consequently, current long-read technologies are unlikely to yield enough sequence information to assemble the genomes of low-abundance species in a metagenome. This issue is compounded by the fact that long-read technologies typically have higher per-base error rates than their short-read counterparts, making high coverage or multi-technology approaches necessary for single-base resolution of microbial genomes [1,101]. Single-cell sequencing has been used to target specific species within a complex community [102,103]. In this technique, single cells are separated from a mixture by micromanipulations. To generate adequate DNA from a single genome copy, it is necessary to amplify the initial sample by a highly processive polymerase in a process called multiple displacement amplification (MDA) [104]. Although this technique is useful for targeted sequencing of specific low abundance organisms, it is inherently low throughput and therefore not suitable for untargeted searches. MDA also tends to produce amplification artifacts causing uneven genome coverage [56]. Moreover, because the amount of input template for MDA is very low, the technique is highly vulnerable to contamination from extracellular DNA originating from other species [105,106].

### 4.2. Metagenomic Binning and Practical Considerations for BGC Analysis

Given the practical limitations of sequencing technologies available for metagenomic studies, *de novo* assemblies are often unable to reconstruct complete microbial chromosomes from metagenomic samples. However, strain-level resolution of complex metagenomes is an important goal for the greater understanding of microbial ecology, including the inter-species interactions mediated by natural products. For instance, understanding which uncultured species produces a given natural product can facilitate attempts to target this species for axenic culture [107,108,109,110,111]. Alternatively, such knowledge enables one to recreate a BGC of interest using synthetic biology and heterologous expression [59,112,113], particularly when a BGC and/or its regulatory elements and resistance genes are fragmented throughout the genome. Thus, access to genome-level resolution motivates the practice of “binning”, or assigning contigs assembled from a metagenomic assembly to discrete genomic entities. Indeed, many groups have devised creative approaches that involve grouping contigs based on sequence composition, coverage, and homology or combinations thereof [114,115,116,117,118,119,120,121,122,123,124,125,126,127,128,129,130,131,132]. However, to accurately interpret the results of metagenomic binning, it is important to be aware of the assumptions and limitations of each of these strategies.

Binning programs that rely heavily or entirely on taxonomic classification [126,133] suffer from their inability to characterize bacteria that diverge significantly from reference genomes [134]. Given that most uncultured bacteria lack high quality reference genome sequences [49], this represents a major limitation. Composition-based binning relies on the principle that the frequency of short oligonucleotide sequences (i.e., “*k*-mers”) throughout a given microbial genome differs between microbial species [135,136,137]. Thus, this method of separating metagenomic sequences into discrete genome bins does not require any previous knowledge of taxonomy, and therefore does not rely on reference databases. However, composition-based binning is only effective with high quality genome assemblies with relatively long contigs (>1000 bp). It is also fundamentally based on the assumption that sequence composition is consistent throughout a given genome, which, of course, is not always the case. Large portions of bacterial genomes, including BGCs, can be transmitted horizontally, and thus can have sequence characteristics that diverge substantially from certain conserved core sequences, such as those associated with protein synthesis or DNA repair. In the case of “*Ca.* E. faulkneri”, the patellazoles producer [18,63], intergenic regions were found to have vastly different GC content compared to coding and RNA genes, meaning that binning would have been challenging if assembly quality had been lower. In general, bacteria are well known to have “flexible” genome regions that can vary dramatically even between strains [138,139]. Finally, differential abundance-based binning uses abundance patterns across a given set of samples to assign groups of contigs to genome bins [124]. Although this method may be particularly useful in identifying sequences shared by the same genome that diverge in nucleotide composition (such as a BGC that was acquired via horizontal transmission), it can break down when a co-varying organism’s genome contains significant sequence variants or other types of genetic heterogeneity across multiple samples [140]. The technique also relies on species being present in multiple samples and does not directly help bin genomes unique to single samples, except for allowing the subtraction of shared contigs. An obvious practical limitation of the differential abundance method is the higher cost associated with sequencing multiple samples. As we are often quite sample limited in the natural products field, collecting multiple samples that contain a targeted organism or natural product may not be feasible, depending on the system at hand.

From the standpoint of BGC analysis, the advantages and disadvantages of each type of binning method have a number of practical ramifications. For instance, considering the possibility that a BGC could be fragmented across a chromosome [17,62,63], correctly identifying all of the associated components of such a fragmented BGC can be very challenging without accurate genome binning. Additionally, if BGCs are acquired through recent horizontal transmissions, their nucleotide compositions may diverge from the rest of a given microorganism’s genomic content. Accordingly, BGCs may be mis-assigned or unassigned to genome bins by automated binning programs. These mis-assignments are especially likely if the BGCs in question are fragmented over their repeat regions by *de novo* genome assembly and are not flanked by any other sequences containing stronger phylogenetic markers. Thus, a hybrid binning approach leveraging sequence composition, abundance, and homology is likely to yield the best results for the analysis of BGCs from shotgun metagenomes.

### 4.3. Strategies to Test and Improve the Accuracy and Contiguity of Assemblies and Pathways

The more contiguous a genomic assembly is, the easier it is to bin and analyze any assembled BGCs. However, for *de novo* assembly algorithms, there is typically a trade-off between increased contiguity and rates of misassembly [141,142]. The same extensive repeat regions in some BGCs that can lead to fragmented assemblies can alternatively lead to misassemblies. These misassemblies can, in turn, skew the interpretation of the biosynthetic logic and, by extension, any attempts to interrogate, recreate, or engineer their biochemistry through heterologous expression efforts or synthetic biology. Thus, it is important to critically assess the outputs of these tools and to be aware of appropriate validation techniques. For short read sequencing, manual examination of differential genomic read coverage, for instance, can provide clues into possible misassemblies. For instance, areas of vastly different or abruptly different coverage regions on the same contig might suggest either the joining of a repeat to a non-repeat region of the same genome, or else the chimeric assembly of sequence regions from two genomes with different abundances. A number of open source programs, such as QUAST [141,142], REAPR [143], and Pilon [144], aim to automate the task of identifying misassemblies. REAPR, for instance, can leverage information provided by paired-end reads and large insert sizes (≥1000 bp) to predict assembly errors without the use of a reference genome. These predictions are achieved using the alignments of paired-end reads to *de novo* assemblies and base-by-base statistical analysis (fragment coverage distribution) to predict substitutions, insertions, and deletions, as well as structural errors (e.g., scaffolding errors) [143]. However, it is notable that, depending on their assumptions regarding sequence evolution, these programs (particularly those that require a reference genome) can mistake true re-arrangements or sequence variants as misassemblies [58]. Furthermore, these programs may falsely report misassemblies if the reference genomes in currently available databases are, themselves, originally misassembled.

In conjunction with automated computational tools, the paired-end information provided by Illumina sequencing and alignment of reads to contigs enables network analysis to suggest possible connections in genome assemblies fragmented over repeat regions [124]. These suggested connections can then be used to guide the design of PCR experiments (and Sanger sequencing of resulting amplicons) to validate the organization of highly repetitive pathways [17]. However, careful attention must be paid to ensure that any custom-designed PCR primers do not unintentionally fall within the repeat regions. If so, positive amplification results can be ambiguous and misleading. Rather, such primers should be designed to flank the perimeters of these repeats, which can be identified based on the number of times reads align back to the *de novo* assembly [17]. The sequence of resulting PCR amplicons can be validated using a complementary, high-accuracy and/or long read form of sequencing, such as Sanger sequencing. 

Another experimental consideration for improving the contiguity and general quality of *de novo* assemblies from environmental samples deals with sampling strategy [145]. Although pooling samples prior to sequencing can theoretically improve the sequence coverage of a shared species, it may further complicate *de novo* assembly efforts by introducing genetic microdiversity resulting from subtle genomic changes in species common to all pooled samples. Such was the case in the metagenomic deconvolution of the *Bugula neritina* metagenome [140]. Whereas pooled samples of *B. neritina* larvae appeared to harbor the highest level of the bryostatin-producing symbiont, “*Ca.* Endobugula sertula”, the pooled sample resulted in a very poor assembly compared to the larval brooding chambers (ovicells) taken from a single colony of *B. neritina*. Furthermore, whereas differential coverage binning, as described above, is a popular means of metagenomic deconvolution [116,118,119,121,124], this technique may increase the population summing effect of direct metagenomic sequencing [146] while also overlooking the most interesting organisms in a metagenome if they only appear in one sample [140], and, by the same principle, an interesting biosynthetic pathway if it appears in only one strain.

Another approach to improve the contiguity of *de novo* assemblies is to use combinations of high accuracy short-read technology, with lower accuracy long-read technology. In fact, even with cultured isolates for which PacBio sequencing is tractable, Illumina sequencing is often included for error correction and variant calling (to detect SNPs, for instance) [1,2]. This hybrid methodological approach is becoming increasingly popular for metagenomic studies as well [101,147]. A similar approach has been achieved using TruSeq Synthetic Long Reads, which employs a special Illumina sequencing method to achieve multi-kbp reads that can then be complemented with standard paired-end short-read lengths [148]. In addition to scaffolding with sequencing technologies, there are other complementary technologies that have been applied to achieve strain-level resolution from shotgun metagenomes using spatial co-localization techniques, collectively termed Chromosomal Conformation Capture (3C) [149,150,151,152,153,154]. Such approaches may offer a viable option for connecting a BGC that is fragmented by *de novo* genome assembly and unable to be confidently assigned to a genome with automated binning algorithms. Another option is the use of multiple length insert libraries to improve contiguity, which assembly algorithms and downstream bioinformatics tools, such as Pilon [144], can use to refine and correct *de novo* assemblies.

Ultimately, there are many challenges associated with the metagenomic analysis of BGCs. However, understanding the available computational resources and experimental techniques behind these analyses can dramatically improve the chances for successfully and accurately assembling long and repetitive BGCs *de novo*. Leveraging the paired end information of Illumina sequencing to guide experimental validation efforts, using hybrid library preparation techniques, using physical linkage information, complementing short read with long read technologies, and refining sampling strategy can all play important roles in achieving successful *de novo* assembly of genomes and biosynthetic pathways from uncultured bacteria. 

### 4.4. Challenges, Opportunities, and Parallels to Chemical Analysis in Product Structure Prediction through Bioinformatics

Once genomes have been binned and further scaffolded, there remain a number of challenges associated with biosynthetic analysis, and different types of BGCs present different assembly and analysis challenges. For instance, although NRPS and PKS pathways are co-linear with the backbone structure of the small molecules they encode [155], they can contain repeat regions that are orders of magnitude larger than the short read lengths of Illumina sequencing. As described above, these repeat regions often result in fragmentation of *de novo* assemblies. Shorter, less repetitive BGCs that do assemble well *de novo*, such as aminoglycoside (AMG) pathways, often have substrate specificities and products that are difficult to predict bioinformatically, due, in large part, to the lack of sufficient experimental characterization data. For example, little is known about the substrate specificities and tolerances of glycosyltransferases [156], which can complicate or prevent rational structure prediction of AMGs from sequence information. Thus, the sequence characteristics of BGCs present an interesting paradox for BGC assembly and analysis: BGCs that are more difficult to assemble can offer more predictive information of chemical structure, whereas pathways that are easier to assemble offer less information on the structure of the NPs they encode.

The process of assembling pathways or genomes, and resolving repeats, can be thought of as a structure elucidation problem for a linear (DNA) molecule. One must weigh multiple sources of information about local connections to devise an overall solution consistent with all datasets, and be aware of the limitations of each data point. As with structure determination, the true structure is proposed only when all alternate structures are excluded. The general workflow is much like that of characterizing a linear peptide. Genomic read coverage is a marker for abundance, much like integration on a proton NMR spectrum, which can distinguish unique proton signals from multiple overlapping ones (or repeats, in DNA assembly). Paired-end read alignment between contigs is similar to nuclear Overhauser effect spectroscopy/rotating frame nuclear Overhauser effect spectroscopy (NOESY/ROESY) information—indicating that two substructures might be close together. However, just as a ROESY crosspeak does not necessarily suggest a direct short bonding between two substructures, paired-end alignment is based upon the alignment of very short reads, which may or may not be unique to the region of interest. PCR amplification between contigs and subsequent Sanger sequencing can be thought of as direct evidence of connection (similar to a heteronuclear multiple bond correlation [HMBC] experiment). However, as mentioned above, one must carefully design the PCR experiment to give a unique and diagnostic amplicon, lest the result be unwittingly ambiguous, similar to an HMBC peak where both carbon and proton signals overlap with other parts of the molecule. 

Although more repetitive in their sequence composition, if NRPS and type I PKS pathways can be adequately (and accurately) assembled, certain useful aspects of product structure can be predicted from these modular pathways. Both of these types of pathways contain large proteins with multiple enzymatic domains, which act like assembly lines and have been extensively reviewed elsewhere [157,158]. In essence, the order of chemical transformations can be deduced from the modular configuration of both NRPS and type 1 *cis*-AT PKS systems, enabling facile prediction of the 2D structure of intermediates that are covalently attached to the PKS/NRPS. The structures of starter and extender units can be predicted from sequence analysis of acyltransferase (AT) [159,160,161] and adenylation (A) [162] domains for PKS and NRPS systems, respectively. The nature of tailoring reactions on these units can be predicted by the presence of certain optional domains in each “module” responsible for adding a unit and elaborating it. This task is somewhat complicated in certain pathways that deviate from co-linearity. The *trans*-AT PKS pathways, where inactive domains, module “skipping” and noncanonical domain orders and locations are common [37,163] present great cases in point. The configuration of some stereocenters can be predicted in both NRPS and PKS systems, and these predictions yield absolute rather than relative configurations, potentially aiding chemical and spectroscopic methods which could elucidate the relative configuration to other centers [5]. 

Despite the useful information provided by genomic analysis and the interesting parallels to chemical analysis, there remain key limitations of structural prediction from biosynthetic pathways. Therefore, it is much easier to assign a biosynthetic pathway (from a list of possible candidates) to a known natural product rather than to predict natural product structures *de novo* from pathways. For instance, the identification of a RiPP pathway for the biosynthesis of the patellamides was inspired by the discovery of a precursor peptide gene containing amino acids in an order consistent with the generation of biosynthetic precursors for patellamides A and C [64]. Conversely, when sequencing a new RiPP pathway, the types of modifications in the final structure can be proposed from the presence of certain genes, but the regiospecificity of these genes cannot typically be predicted, nor can a single, absolute structure be proposed. In modular pathways, although the linear structure (with some stereocenters) produced by a PKS or NRPS system can be predicted, the final structure is often elusive for a number of reasons. Many NRPS and PKS pathways end with a thioesterase (TE) domain that liberates either a linear or cyclic product from the assembly line machinery. The linear/cyclic nature or the site of cyclization cannot be predicted with current sequence-based methods. As with RiPPs, we can often predict the *type* of post-PKS or post-NRPS tailoring reactions that occur from the presence of certain genes in the pathway, but the *nature* and *location* of these reactions are often elusive.

### 4.5. Successful Examples of Repetitive BGCs Analyzed by De Novo Assembly

Despite the many challenges associated with *de novo* BGC analysis, there are a number of studies in the natural products realm demonstrating the balance of computational analysis and experimental design to overcome such challenges. For the purposes of brevity and clarity, we highlight here two studies relevant to marine natural products. 

One such recent example was the targeted assembly of the genome of the uncultured, bryostatin-producing symbiont, “*Ca.* E. sertula”, from the metagenome of its bryozoan host, *B. neritina*. A large section of the *bry* pathway for the biosynthesis of bryostatins had been sequenced through clone library methods by the Sherman and Haygood groups [96,97]. This was a painstaking task as multiple nearly exact, long repeats in the pathway made clones unstable to homologous recombination. The 16S rRNA gene sequence of “*Ca.* E. sertula” was separately found to co-localize with the *bry* pathway, suggesting that the pathway belonged to this symbiont [164]. Later, Illumina shotgun sequencing was used to recover the symbiont’s genome directly from the host tissue in order to glean information on its primary metabolism and to recover missing components of the biosynthetic pathway encoding the bryostatins [17]. 

Due to the large exact repeats in the *bry* pathway, it was similarly challenging to reconstruct with *de novo* assembly using Illumina reads [17]. The first iteration of *de novo* assembly from the shotgun sequencing of *B. neritina* ovicells produced a different arrangement of the *bry* pathway than was reported by Sudek et al. [97]. However, this re-arrangement was ultimately identified as a misassembly resulting from non-optimal assembler parameters, rather than a bona fide re-arrangement or sequence variant, and the original structure of the *bry* pathway suggested by the Sanger sequencing of individual clones [96,97] was validated using a combination of computational and experimental techniques leveraging paired-end read information using a method adapted from Albertsen et al. [17,124]. Both putative arrangements were tested extensively with PCR and Sanger sequencing [17], and only the originally proposed arrangement yielded unambiguous PCR amplification. This work showed that Illumina data could be used to correctly reconstruct a repeat-laden pathway, but it also suggested that vigilance should be applied during assembly, especially where the true sequence is unknown.

This experience highlights the importance of implementing both computational and experimental methods to scrutinize and validate the arrangement of biosynthetic pathways generated via *de novo* assembly. Although untargeted sequencing and *de novo* assembly can present ambiguous or misleading results without proper scrutiny, they also have the potential to uncover new genomic content that would be unseen in reference-based methods and difficult to identify using traditional clone-library methods, due to fragmentation across the chromosome. In addition, because a shotgun sequencing and *de novo* assembly approach was employed in the study of “*Ca.* E. sertula”, previously missing components of the *bry* pathway, as well as a number of deficiencies in the symbiont’s primary metabolism, were successfully identified [17].

The *slm* pathway, in the *Salinispora tropica* genome, represents an early and exemplary case of integrating genomic and chemical analysis to simultaneously resolve repeat-laden biosynthetic architecture and its encoded chemical structure. In 2007, Udwary et al. assembled the *S. tropica* genome and discovered the broad array of biosynthetic potential it contained [165]. One major challenge, however, was resolving the genome into a single circular chromosome due to the highly repetitive nature of the *slm* pathway [165,166]. Elucidation of the salinilactam structure generated by this BGC also proved challenging. After extensive efforts, it was determined that salinilactam is a macrocyclic polyketide bearing one amino acid unit. Often, the configuration of polyketides is challenging to elucidate—relative configurations of adjacent centers can be determined by *J*-based NMR analysis [167], but the relative configurations of separated regions [168] and isolated centers are often elusive. In salinilactam, the exact placement of the methyl group in the southern portion of the molecule was also likely challenging. It is situated in the middle of six unadorned *trans*-double bonds likely to have very similar proton and carbon chemical shifts. 

In the end, the authors were able to integrate spectroscopic and bioinformatic analyses to elucidate both the chemical structure of the salinilactams and the arrangement of the lengthy BGC encoding them [165,166]. The molecular formula, obtained through mass spectrometry, likely helped in the resolution of contigs and repeats in the pathway by unambiguously determining how many PKS and NRPS modules were required for construction of the compound. Likewise, the placement of a module incorporating methylmalonate among four other modules incorporating malonate unambiguously determined the placement of the methyl group in the final molecule. Additionally, bioinformatics was used to propose the absolute configuration of three hydroxyl groups in the molecule. Considering the nature of the salinilactam structure and the sequence characteristics of the *slm* BGC, this resolution represents an impressive achievement. However, such genomic analysis would likely not have been possible if the *S. tropica* had not been isolated and cultured in laboratory settings. This caveat invites an interesting question: is it possible to assemble *de novo* a BGC of *slm*’s length and sequence composition using current short read technology available for metagenomic analysis? How could various sequencing parameters be tuned to improve the *de novo* assembly of such a BGC? 

### 4.6. Analysis of Sequencing Parameters on *slm* Pathway Fragmentation in *Salinispora tropica*

In addressing questions such as those posed above in Section 4.5, we have endeavored to evaluate the impact of various sequencing parameters on the fragmentation of *de novo* assemblies and BGCs in particular. To explore these effects, we simulated Illumina HiSeq shotgun sequencing data with Art Illumina (available at http://www.niehs.nih.gov/research/resources/software/art [169]) and PacBio data with PBSIM (available at https://github.com/pfaucon/PBSIM-PacBio-Simulator [170]), focusing mostly on the effects of read length and depth of sequencing coverage on the fragmentation of BGCs and the genomic assemblies as a whole (see Supplemental Information for more details on the simulation and assembly methods). We chose to use the *Salinospora tropica* genome as it represents the first available genome from a marine-derived natural product producer and it houses a broad diversity of biosynthetic pathways. Additionally, the authors who reported this genome sequence noted significant difficulties during the assembly of the long and highly repetitive salinilactam biosynthetic pathway, which could only be resolved using both computational and chemical data [165,166] (see above). 

**Figure 1:**
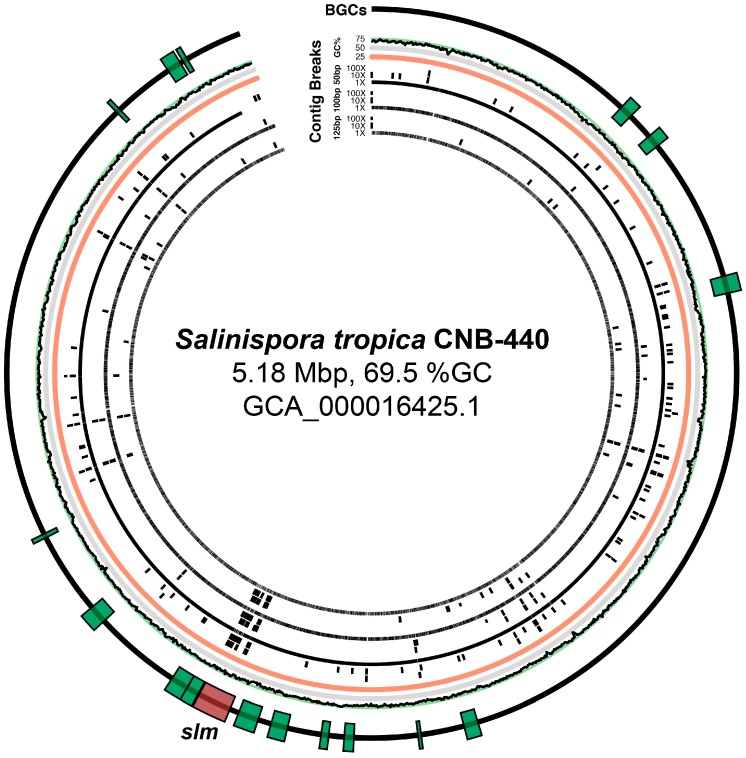
Circular genome map of *de novo* assemblies mapped back to the *Salinospora tropica* CNB-440 reference genome (GCA_0016425.1). Simulated Illumina HiSeq 2500 sequencing data show assembly fragmentation (indicated by black bars) throughout the chromosome, including in BGCs (annotated as green boxes in the outermost ring; the *slm* pathway is annotated in red) using a mean insert size of 275 bp and different combinations of read length (50–125 bp) and sequencing depth (1–100×).

**Figure 2:**
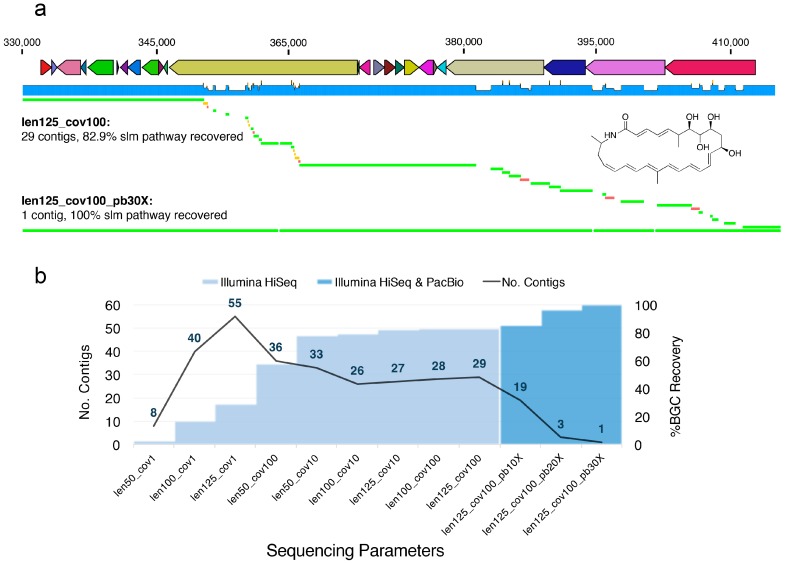
(**a**) Alignment of *de novo* contigs to the reference *slm* pathway. *De novo* contigs colored in green, yellow, and red mapped to the reference *slm* pathway sequence one, two, and three times, respectively. In other words, contigs colored in red mapped to three different locations in the *slm* BGC, due to exact repeats. (**b**) Fragmentation and percent (in length) recovery of the salinilactam biosynthetic gene cluster based on combinations of read length and depth of sequencing on a simulated Illumina HiSeq 2500 platform run with and without PacBio CLR sequencing. An insert size of 275 bp was used for all of 12 simulated sequencing runs displayed here (the results obtained using a longer fragment size and greater sequencing depths are also explored in Figure S1). Notably, 30× PacBio coverage was required to fully scaffold the Illumina-based assembly with a read length of 125 bp and 100× coverage (len125_cov100_pb30×, where the numbers following “len” describes the Illumina read length, “cov” the depth of Illumina read coverage, and “pb” the depth of PacBio coverage).

We found that, even with longer read lengths (125 bp) and higher depths of sequencing (100×), the *S. tropica* genome was heavily fragmented (Table S1), especially in the region containing the *slm* pathway (Figure 1). Thus, we wondered if even greater depths or longer fragment sizes could alleviate the shortcomings of short-read Illumina technology. We postulated that such an approach may also allow scaffolding of the *slm* pathway into fewer contigs than could be achieved with a 100× coverage and an insert size of 275 bp (Figure 2). However, even with 1000× sequence coverage and a longer insert size (1000 bp vs. 275 bp), we were unable to further scaffold the repetitive *slm* pathway (Figure S1 and Table S1). Furthermore, we found that increased sequencing depth correlated to greater genome assembly fragmentation in some cases; this was especially pronounced for certain shorter read lengths (50 bp), particularly inside of the *slm* BGC (Figure 1 and Figure 2). Interestingly, for the simulations of the sequencing with the shortest read length (50 bp), the low-intermediate (10×) coverage provided the best assembly in terms of percent of the *slm* BGC recovered (Figure 2), but not general genome statistics such as N_50_ and contiguity (Figure S2 and Table S1).

Scientists may assume that greater sequencing coverage typically provides higher quality assemblies, and thus, may end up paying more to achieve greater sequencing depth. However, consistent with our exploratory analysis here, some studies suggest that greater depth does not necessarily afford more contiguous assemblies and that ultra-deep sequencing (>1000× coverage) may actually be counterproductive if not explicitly handled using specialized assembly algorithms [171,172], due, in part, to the amplification of read duplication events and other sequencing errors [56]. From a practical standpoint, even if pre-processing steps successfully address the issues associated with ultra-deep sequencing, the significantly compounded cost of 10× more sequencing depth may not translate to greater information.

The simulations and analysis approaches presented here are not meant to be exhaustive nor are they intended to suggest fundamental principles. Rather, our discussion of them is intended to encourage other natural product scientists to think carefully about the most appropriate sampling strategy, sequencing parameters or platforms, as well as to remain wary of the potential pitfalls surrounding the *de novo* assembly process. The perspectives outlined here are intended to highlight the importance of leveraging all available tools, be they computational or experimental, at researchers’ disposal to interrogate the results of this process. Ultimately, we were not able to assemble the *slm* BGC using any feasible combination of read length, sequencing depth, or fragment size on a simulated Illumina HiSeq platform, which remains the most relevant platform for *de novo* metagenomic assembly. The best combination of parameters for Illumina simulation alone was a read length of 125 bp, 100× coverage, and a fragment size of 275 bp (Figure 2). This set of parameters recovered 82.9% of the pathway in 29 contigs (Figure 2). We were only able to resolve the pathway into a single contiguous sequence using 30× coverage with PacBio sequencing (in addition to Illumina sequencing), which is not practically feasible for most metagenomic applications, due to cost and difficulties involved in obtaining DNA of high enough quality (see above). However, these simulations further suggest the value of using multiple sequencing technologies and the importance of integrating chemical and computational techniques, as exemplified by Udwary et al. [165], to resolve challenging problems in BGC analysis.

Sequencing [1] and bioinformatics [54,173,174,175,176,177] have come a long way and continue to revolutionize the field of natural products drug discovery. However, the information offered by these techniques is not infallible and should not be unequivocally interpreted in the vacuum of purely computational analysis. Although long read technology platforms are currently not economically feasible for generating the sequencing depth required for metagenomic *de novo* assembly, we anticipate that as the throughput, accuracy, and price continue to improve, the contiguity of these difficult to assemble BGCs could be dramatically improved. For now, however, it remains crucial to be cautious and skeptical [178,179] when assembling and interpreting large and repetitive biosynthetic pathways. Any assembly of a BGC pathway should be examined and validated experimentally or with complementary sequencing technologies, such as Sanger, PacBio, or Oxford Nanopore, where possible. To do otherwise, could result in misleading and inaccurate interpretations of the mechanics and evolutionary history of these pathways that offer great promise in providing a continuing supply of novel, bioactive compounds.

## 5. Conclusions

Current sequencing, assembly, and binning methods used to investigate BGCs have a number of notable strengths and weaknesses. Although these methods are powerful, allowing the investigation of BGCs even from uncultured sources, it should be apparent from this article that potential complications need to be taken into account and are context dependent. Consequently, there are no bioinformatic panaceas for BGC assembly and analysis. Researchers should therefore treat the output of bioinformatic applications with healthy skepticism, just as they should question and independently verify the results of instrumental measurements (e.g., complementing NMR with mass spectrometry analysis). There are a number of problems that do not yet have completely generalizable solutions in BGC analysis and metagenomics. A fundamental problem is that total structure prediction from cluster sequence is not yet possible; this clearly complicates the task of genome mining. Efforts have been made to collate and standardize the annotation of BGCs [180], which could aid future efforts to improve structure prediction. Another problem is that metagenomic binning is still difficult, often requiring much manual data processing and effort, a significant barrier for entry for groups interested in shotgun metagenomic sequencing. On the experimental side, there are two roadblocks contributing to the supply problem for any natural product made by an uncultured organism. The first of these entails the difficulty in culturing the majority of environmental microbes. It may well be possible to culture more environmental microbes than previously thought [44,46], but finding appropriate culturing conditions that are both selective and specific is a significant challenge. A potential solution to this challenge may lie in improved automatic annotation and metabolic modeling [181,182] of genomes obtained through metagenomics to predict growth rates and conditions. The other major problem is that heterologous expression is challenging, especially for large pathways, such as PKS and NRPS systems, and for pathways originating from uncultured organisms. Such pathways will likely not be suited to heterologous hosts, requiring *de novo* synthesis and refactoring [74] to provide optimal codon usage and compatible promoters, respectively. Advances in synthetic biology may ultimately alleviate this challenge but rational methods to identify and correct expression problems will still be needed. Ultimately, much has been achieved in developing tools to accurately correlate genomic information to structural information when it comes to natural products biosynthesis. However, this area of study continues to be heavily investigated and promises to provide challenging and rewarding work for years to come. 

## Supplementary Materials

The following are available online at www.mdpi.com/1660-3397/15/6/165/s1, Supplemental Methods, Figure S1: Fragmentation (no. contigs) and percent recovery of the salinilactm (*slm*) biosynthetic gene cluster based on expanded set of simulated Illumina and PacBio sequencing parameters, Figure S2: (a) Nx (where x is 0–100% of the assembly length) and (b) cumulative length plots for assemblies using a read length of 50 bp, insert size of 275 bp and ranges of sequencing depth (1–1000×), Table S1: Quantitative data on *Salinispora tropica* genome statistics based on simulated sequencing parameters.

## References

[1] Loman N.J., Pallen M.J. (2015). Twenty years of bacterial genome sequencing. Nat. Rev. Microbiol..

[2] Bashir A., Klammer A.A., Robins W.P., Chin C.-S., Webster D., Paxinos E., Hsu D., Ashby M., Wang S., Peluso P. (2012). A hybrid approach for the automated finishing of bacterial genomes. Nat. Biotechnol..

[3] Chin C.-S., Alexander D.H., Marks P., Klammer A.A., Drake J., Heiner C., Clum A., Copeland A., Huddleston J., Eichler E.E. (2013). Nonhybrid, finished microbial genome assemblies from long-read SMRT sequencing data. Nat. Methods.

[4] Lewin G.R., Carlos C., Chevrette M.G., Horn H.A., McDonald B.R., Stankey R.J., Fox B.G., Currie C.R. (2016). Evolution and ecology of Actinobacteria and their bioenergy applications. Annu. Rev. Microbiol..

[5] Adnani N., Ellis G.A., Wyche T.P., Bugni T.S., Kwan J.C., Schmidt E.W. (2014). Emerging trends for stimulating the discovery of natural products. Natural Products Analysis.

[6] Mak S., Xu Y., Nodwell J.R. (2014). The expression of antibiotic resistance genes in antibiotic-producing bacteria. Mol. Microbiol..

[7] Tang X., Li J., Millán-Aguiñaga N., Zhang J.J., O’Neill E.C., Ugalde J.A., Jensen P.R., Mantovani S.M., Moore B.S. (2015). Identification of thiotetronic acid antibiotic biosynthetic pathways by target-directed genome mining. ACS Chem. Biol..

[8] Hagen A., Poust S., de Rond T., Fortman J.L., Katz L., Petzold C.J., Keasling J.D. (2016). Engineering a polyketide synthase for in vitro production of adipic acid. ACS Synth. Biol..

[9] Phelan R.M., Sekurova O.N., Keasling J.D., Zotchev S.B. (2015). Engineering terpene biosynthesis in *Streptomyces* for production of the advanced biofuel precursor bisabolene. ACS Synth. Biol..

[10] Yamanaka K., Reynolds K.A., Kersten R.D., Ryan K.S., Gonzalez D.J., Nizet V., Dorrestein P.C., Moore B.S. (2014). Direct cloning and refactoring of a silent lipopeptide biosynthetic gene cluster yields the antibiotic taromycin A. Proc. Natl. Acad. Sci. USA.

[11] Dias O., Rocha M., Ferreira E.C., Rocha I. (2015). Reconstructing genome-scale metabolic models with merlin. Nucleic Acids Res..

[12] Medema M.H., Blin K., Cimermancic P., de Jager V., Zakrzewski P., Fischbach M.A., Weber T., Takano E., Breitling R. (2011). antiSMASH: Rapid identification, annotation and analysis of secondary metabolite biosynthesis gene clusters in bacterial and fungal genome sequences. Nucleic Acids Res..

[13] Blin K., Wolf T., Chevrette M.G., Lu X., Schwalen C.J., Kautsar S.A., Suarez Duran H.G., de los Santos E.L.C., Kim H.U., Nave M. (2017). antiSMASH 4.0—Improvements in chemistry prediction and gene cluster boundary identification. Nucleic Acids Res..

[14] Weimann A., Mooren K., Frank J., Pope P.B., Bremges A., McHardy A.C. (2016). From genomes to phenotypes: Traitar, the microbial trait analyzer. mSystems.

[15] Bennett G.M., Moran N.A. (2015). Heritable symbiosis: The advantages and perils of an evolutionary rabbit hole. Proc. Natl. Acad. Sci. USA.

[16] McCutcheon J.P., Moran N.A. (2012). Extreme genome reduction in symbiotic bacteria. Nat. Rev. Microbiol..

[17] Miller I.J., Vanee N., Fong S.S., Lim-Fong G.E., Kwan J.C. (2016). Lack of overt genome reduction in the bryostatin-producing bryozoan symbiont “*Candidatus* Endobugula sertula”. Appl. Environ. Microbiol..

[18] Kwan J.C., Schmidt E.W. (2013). Bacterial endosymbiosis in a chordate host: Long-term co-evolution and conservation of secondary metabolism. PLoS ONE.

[19] Medema M.H., Cimermancic P., Sali A., Takano E., Fischbach M.A. (2014). A systematic computational analysis of biosynthetic gene cluster evolution: Lessons for engineering biosynthesis. PLoS Comput. Biol..

[20] Shi Y., Tyson G.W., Eppley J.M., DeLong E.F. (2011). Integrated metatranscriptomic and metagenomic analyses of stratified microbial assemblages in the open ocean. ISME J..

[21] Haq I.U., van Elsas I.J.D., Zeilinger S., Martín J.-F., García-Estrada C. (2015). Metagenomics and metatranscriptomics for the exploration of natural products from soil fungi. Biosynthesis and Molecular Genetics of Fungal Secondary Metabolites, Volume 2.

[22] Clardy J., Fischbach M.A., Currie C.R. (2009). The natural history of antibiotics. Curr. Biol..

[23] Ream D.C., Bankapur A.R., Friedberg I. (2015). An event-driven approach for studying gene block evolution in bacteria. Bioinformatics.

[24] Johnson S.S., Chevrette M.G., Ehlmann B.L., Benison K.C. (2015). Insights from the metagenome of an acid salt lake: The role of biology in an extreme depositional environment. PLoS ONE.

[25] Choi H., Oh D.C. (2015). Considerations of the chemical biology of microbial natural products provide an effective drug discovery strategy. Arch. Pharm. Res..

[26] Flórez L.V., Biedermann P.H.W., Engl T., Kaltenpoth M. (2015). Defensive symbioses of animals with prokaryotic and eukaryotic microorganisms. Nat. Prod. Rep..

[27] Ramadhar T.R., Beemelmanns C., Currie C.R., Clardy J. (2014). Bacterial symbionts in agricultural systems provide a strategic source for antibiotic discovery. J. Antibiot..

[28] Chevrette M.G., Aicheler F., Kohlbacher O., Currie C.R., Medema M.H. SANDPUMA: Ensemble predictions of nonribosomal peptide chemistry reveals biosynthetic diversity across Actinobacteria. Bioinformatics.

[29] Calteau A., Fewer D.P., Latifi A., Coursin T., Laurent T., Jokela J., Kerfeld C.A., Sivonen K., Piel J., Gugger M. (2014). Phylum-wide comparative genomics unravel the diversity of secondary metabolism in Cyanobacteria. BMC Genom..

[30] Cimermancic P., Medema M.H., Claesen J., Kurita K., Wieland Brown L.C., Mavrommatis K., Pati A., Godfrey P.A., Koehrsen M., Clardy J. (2014). Insights into secondary metabolism from a global analysis of prokaryotic biosynthetic gene clusters. Cell.

[31] Cruz-Morales P., Martínez-Guerrero C.E. (2016). Phylogenomic analysis of natural products biosynthetic gene clusters allows discovery of arseno-organic metabolites in model Streptomycetes. Genome Biol. Evol..

[32] Zucko J., Cullum J., Hranueli D., Long P.F. (2011). Evolutionary dynamics of modular polyketide synthases, with implications for protein design and engineering. J. Antibiot..

[33] Jenke-Kodama H., Dittmann E. (2009). Evolution of metabolic diversity: Insights from microbial polyketide synthases. Phytochemistry.

[34] Rounge T.B., Rohrlack T., Kristensen T., Jakobsen K.S. (2008). Recombination and selectional forces in cyanopeptolin NRPS operons from highly similar, but geographically remote *Planktothrix* strains. BMC Microbiol..

[35] Rausch C., Hoof I., Weber T., Wohlleben W., Huson D.H. (2007). Phylogenetic analysis of condensation domains in NRPS sheds light on their functional evolution. BMC Evol. Biol..

[36] Jenke-Kodama H., Börner T., Dittmann E. (2006). Natural biocombinatorics in the polyketide synthase genes of the actinobacterium *Streptomyces avermitilis*. PLoS Comput. Biol..

[37] Helfrich E.J.N., Piel J. (2016). Biosynthesis of polyketides by *trans*-AT polyketide synthases. Nat. Prod. Rep..

[38] Nguyen D.D., Melnik A.V., Koyama N., Lu X., Schorn M., Fang J., Aguinaldo K., Lincecum T.L., Ghequire M.G.K., Carrion V.J. (2016). Indexing the *Pseudomonas* specialized metabolome enabled the discovery of poaeamide B and the bananamides. Nat. Microbiol..

[39] Yang X., van der Donk W.A. (2013). Ribosomally synthesized and post-translationally modified peptide natural products: New insights into the role of leader and core peptides during biosynthesis. Chemistry.

[40] Sardar D., Pierce E., McIntosh J.A., Schmidt E.W. (2015). Recognition sequences and substrate evolution in cyanobactin biosynthesis. ACS Synth. Biol..

[41] Doroghazi J.R., Albright J.C., Goering A.W., Ju K.-S., Haines R.R., Tchalukov K.A., Labeda D.P., Kelleher N.L., Metcalf W.W. (2014). A roadmap for natural product discovery based on large-scale genomics and metabolomics. Nat. Chem. Biol..

[42] Staley J.T., Konopka A. (1985). Measurement of *in situ* activities of nonphotosynthetic microorganisms in aquatic and terrestrial habitats. Annu. Rev. Microbiol..

[43] Browne H.P., Forster S.C., Anonye B.O., Kumar N., Neville B.A., Stares M.D., Goulding D., Lawley T.D. (2016). Culturing of “unculturable” human microbiota reveals novel taxa and extensive sporulation. Nature.

[44] Ling L.L., Schneider T., Peoples A.J., Spoering A.L., Engels I., Conlon B.P., Mueller A., Schäberle T.F., Hughes D.E., Epstein S. (2015). A new antibiotic kills pathogens without detectable resistance. Nature.

[45] Lok C. (2015). Mining the microbial dark matter. Nature.

[46] Stewart E.J. (2012). Growing unculturable bacteria. J. Bacteriol..

[47] Yarza P., Yilmaz P., Pruesse E., Glöckner F.O., Ludwig W., Schleifer K.-H., Whitman W.B., Euzéby J., Amann R., Rosselló-Móra R. (2014). Uniting the classification of cultured and uncultured bacteria and archaea using 16S rRNA gene sequences. Nat. Rev. Microbiol..

[48] Brown C.T., Hug L.A., Thomas B.C., Sharon I., Castelle C.J., Singh A., Wilkins M.J., Wrighton K.C., Williams K.H., Banfield J.F. (2015). Unusual biology across a group comprising more than 15% of domain Bacteria. Nature.

[49] Rinke C., Schwientek P., Sczyrba A., Ivanova N.N., Anderson I.J., Cheng J.-F., Darling A., Malfatti S., Swan B.K., Gies E.A. (2013). Insights into the phylogeny and coding potential of microbial dark matter. Nature.

[50] Rath C.M., Janto B., Earl J., Ahmed A., Hu F.Z., Hiller L., Dahlgren M., Kreft R., Yu F., Wolff J.J. (2011). Meta-omic characterization of the marine invertebrate microbial consortium that produces the chemotherapeutic natural product ET-743. ACS Chem. Biol..

[51] Escobar-Zepeda A., Vera-Ponce de León A., Sanchez-Flores A. (2015). The road to metagenomics: From microbiology to DNA sequencing technologies and bioinformatics. Front. Genet..

[52] Edlund A., Loesgen S., Fenical W., Jensen P.R. (2011). Geographic distribution of secondary metabolite genes in the marine actinomycete *Salinispora arenicola*. Appl. Environ. Microbiol..

[53] Ziemert N., Lechner A., Wietz M., Millán-Aguiñaga N., Chavarria K.L., Jensen P.R. (2014). Diversity and evolution of secondary metabolism in the marine actinomycete genus *Salinispora*. Proc. Natl. Acad. Sci. USA.

[54] Mick E., Sorek R. (2014). High-resolution metagenomics. Nat. Biotechnol..

[55] Compeau P.E.C., Pevzner P.A., Tesler G. (2011). How to apply de Bruijn graphs to genome assembly. Nat. Biotechnol..

[56] Bankevich A., Nurk S., Antipov D., Gurevich A.A., Dvorkin M., Kulikov A.S., Lesin V.M., Nikolenko S.I., Pham S., Prjibelski A.D. (2012). SPAdes: A new genome assembly algorithm and its applications to single-cell sequencing. J. Comput. Biol..

[57] Cleary B., Brito I.L., Huang K., Gevers D., Shea T., Young S., Alm E.J. (2015). Detection of low-abundance bacterial strains in metagenomic datasets by eigengenome partitioning. Nat. Biotechnol..

[58] Nurk S., Meleshko D., Korobeynikov A., Pevzner P.A. (2017). metaSPAdes: A new versatile metagenomic assembler. Genome Res..

[59] Chang F.-Y., Ternei M.A., Calle P.Y., Brady S.F. (2015). Targeted metagenomics: Finding rare tryptophan dimer natural products in the environment. J. Am. Chem. Soc..

[60] Kang H.-S., Brady S.F. (2014). Arixanthomycins A-C: Phylogeny-guided discovery of biologically active eDNA-derived pentangular polyphenols. ACS Chem. Biol..

[61] Freeman M.F., Gurgui C., Helf M.J., Morinaka B.I., Uria A.R., Oldham N.J., Sahl H.-G., Matsunaga S., Piel J. (2012). Metagenome mining reveals polytheonamides as posttranslationally modified ribosomal peptides. Science.

[62] Schofield M.M., Jain S., Porat D., Dick G.J., Sherman D.H. (2015). Identification and analysis of the bacterial endosymbiont specialized for production of the chemotherapeutic natural product ET-743. Environ. Microbiol..

[63] Kwan J.C., Donia M.S., Han A.W., Hirose E., Haygood M.G., Schmidt E.W. (2012). Genome streamlining and chemical defense in a coral reef symbiosis. Proc. Natl. Acad. Sci. USA.

[64] Schmidt E.W., Nelson J.T., Rasko D.A., Sudek S., Eisen J.A., Haygood M.G., Ravel J. (2005). Patellamide A and C biosynthesis by a microcin-like pathway in *Prochloron didemni*, the cyanobacterial symbiont of *Lissoclinum patella*. Proc. Natl. Acad. Sci. USA.

[65] Nakabachi A., Ueoka R., Oshima K., Teta R., Mangoni A., Gurgui M., Oldham N.J., van Echten-Deckert G., Okamura K., Yamamoto K. (2013). Defensive bacteriome symbiont with a drastically reduced genome. Curr. Biol..

[66] Kampa A., Gagunashvili A.N., Gulder T.A.M., Morinaka B.I., Daolio C., Godejohann M., Miao V.P.W., Piel J., Andrésson Ó.S. (2013). Metagenomic natural product discovery in lichen provides evidence for a family of biosynthetic pathways in diverse symbioses. Proc. Natl. Acad. Sci. USA.

[67] Piel J., Wen G., Platzer M., Hui D. (2004). Unprecedented diversity of catalytic domains in the first four modules of the putative pederin polyketide synthase. Chembiochem.

[68] Piel J. (2002). A polyketide synthase-peptide synthetase gene cluster from an uncultured bacterial symbiont of *Paederus* beetles. Proc. Natl. Acad. Sci. USA.

[69] Fisch K.M., Gurgui C., Heycke N., van der Sar S.A., Anderson S.A., Webb V.L., Taudien S., Platzer M., Rubio B.K., Robinson S.J. (2009). Polyketide assembly lines of uncultivated sponge symbionts from structure-based gene targeting. Nat. Chem. Biol..

[70] Piel J., Hui D., Wen G., Butzke D., Platzer M., Fusetani N., Matsunaga S. (2004). Antitumor polyketide biosynthesis by an uncultivated bacterial symbiont of the marine sponge *Theonella swinhoei*. Proc. Natl. Acad. Sci. USA.

[71] Donia M.S., Cimermancic P., Schulze C.J., Wieland Brown L.C., Martin J., Mitreva M., Clardy J., Linington R.G., Fischbach M.A. (2014). A systematic analysis of biosynthetic gene clusters in the human microbiome reveals a common family of antibiotics. Cell.

[72] Guo C.-J., Chang F.-Y., Wyche T.P., Backus K.M., Acker T.M., Funabashi M., Taketani M., Donia M.S., Nayfach S., Pollard K.S. (2017). Discovery of reactive microbiota-derived metabolites that inhibit host proteases. Cell.

[73] Vila-Farres X., Chu J., Inoyama D., Ternei M.A., Lemetre C., Cohen L.J., Cho W., Reddy B.V.B., Zebroski H.A., Freundlich J.S. (2017). Antimicrobials inspired by nonribosomal peptide synthetase gene clusters. J. Am. Chem. Soc..

[74] Smanski M.J., Bhatia S., Zhao D., Park Y., Woodruff L.B.A., Giannoukos G., Ciulla D., Busby M., Calderon J., Nicol R. (2014). Functional optimization of gene clusters by combinatorial design and assembly. Nat. Biotechnol..

[75] Smanski M.J., Zhou H., Claesen J., Shen B., Fischbach M.A., Voigt C.A. (2016). Synthetic biology to access and expand nature’s chemical diversity. Nat. Rev. Microbiol..

[76] Iqbal H.A., Low-Beinart L., Obiajulu J.U., Brady S.F. (2016). Natural product discovery through improved functional metagenomics in *Streptomyces*. J. Am. Chem. Soc..

[77] Kelsic E.D., Zhao J., Vetsigian K., Kishony R. (2015). Counteraction of antibiotic production and degradation stabilizes microbial communities. Nature.

[78] Wright E.S., Vetsigian K.H. (2016). Inhibitory interactions promote frequent bistability among competing bacteria. Nat. Commun..

[79] Brady S.F., Chao C.J., Handelsman J., Clardy J. (2001). Cloning and heterologous expression of a natural product biosynthetic gene cluster from eDNA. Org. Lett..

[80] Wang G.Y., Graziani E., Waters B., Pan W., Li X., McDermott J., Meurer G., Saxena G., Andersen R.J., Davies J. (2000). Novel natural products from soil DNA libraries in a streptomycete host. Org. Lett..

[81] Bok J.W., Ye R., Clevenger K.D., Mead D., Wagner M., Krerowicz A., Albright J.C., Goering A.W., Thomas P.M., Kelleher N.L. (2015). Fungal artificial chromosomes for mining of the fungal secondary metabolome. BMC Genom..

[82] Craig J.W., Cherry M.A., Brady S.F. (2011). Long-chain *N*-acyl amino acid synthases are linked to the putative PEP-CTERM/exosortase protein-sorting system in Gram-negative bacteria. J. Bacteriol..

[83] Cohen L.J., Kang H.-S., Chu J., Huang Y.-H., Gordon E.A., Reddy B.V.B., Ternei M.A., Craig J.W., Brady S.F. (2015). Functional metagenomic discovery of bacterial effectors in the human microbiome and isolation of commendamide, a GPCR G2A/132 agonist. Proc. Natl. Acad. Sci. USA.

[84] Piel J. (2009). Metabolites from symbiotic bacteria. Nat. Prod. Rep..

[85] Salvador-Reyes L.A., Engene N., Paul V.J., Luesch H. (2015). Targeted natural products discovery from marine cyanobacteria using combined phylogenetic and mass spectrometric evaluation. J. Nat. Prod..

[86] Shih P.M., Matzke N.J. (2013). Primary endosymbiosis events date to the later Proterozoic with cross-calibrated phylogenetic dating of duplicated ATPase proteins. Proc. Natl. Acad. Sci. USA.

[87] Kamel L., Keller-Pearson M., Roux C., Ané J.-M. (2017). Biology and evolution of arbuscular mycorrhizal symbiosis in the light of genomics. New Phytol..

[88] Smanski M.J., Schlatter D.C., Kinkel L.L. (2016). Leveraging ecological theory to guide natural product discovery. J. Ind. Microbiol. Biotechnol..

[89] Yu T.-W., Bai L., Clade D., Hoffmann D., Toelzer S., Trinh K.Q., Xu J., Moss S.J., Leistner E., Floss H.G. (2002). The biosynthetic gene cluster of the maytansinoid antitumor agent ansamitocin from *Actinosynnema pretiosum*. Proc. Natl. Acad. Sci. USA.

[90] Piel J., Höfer I., Hui D. (2004). Evidence for a symbiosis island involved in horizontal acquisition of pederin biosynthetic capabilities by the bacterial symbiont of *Paederus fuscipes* beetles. J. Bacteriol..

[91] Moran N.A., Munson M.A., Baumann P., Ishikawa H. (1993). A molecular clock in endosymbiotic bacteria is calibrated using the insect hosts. Proc. R. Soc. Lond. B Biol. Sci..

[92] Tamas I., Klasson L., Canbäck B., Näslund A.K., Eriksson A.-S., Wernegreen J.J., Sandström J.P., Moran N.A., Andersson S.G.E. (2002). 50 million years of genomic stasis in endosymbiotic bacteria. Science.

[93] Trindade-Silva A.E., Lim-Fong G.E., Sharp K.H., Haygood M.G. (2010). Bryostatins: Biological context and biotechnological prospects. Curr. Opin. Biotechnol..

[94] Lim-Fong G.E., Regali L.A., Haygood M.G. (2008). Evolutionary relationships of “*Candidatus* endobugula” bacterial symbionts and their *Bugula* bryozoan hosts. Appl. Environ. Microbiol..

[95] Fehlauer-Ale K.H., Mackie J.A., Lim-Fong G.E., Ale E., Pie M.R., Waeschenbach A. (2014). Cryptic species in the cosmopolitan *Bugula neritina* complex (Bryozoa, Cheilostomata). Zool. Scr..

[96] Hildebrand M., Waggoner L.E., Liu H., Sudek S., Allen S., Anderson C., Sherman D.H., Haygood M. (2004). *bryA*: An unusual modular polyketide synthase gene from the uncultivated bacterial symbiont of the marine bryozoan Bugula neritina. Chem. Biol..

[97] Sudek S., Lopanik N.B., Waggoner L.E., Hildebrand M., Anderson C., Liu H., Patel A., Sherman D.H., Haygood M.G. (2007). Identification of the putative bryostatin polyketide synthase gene cluster from “*Candidatus* Endobugula sertula”, the uncultivated microbial symbiont of the marine bryozoan *Bugula neritina*. J. Nat. Prod..

[98] Sit C.S., Ruzzini A.C., Van Arnam E.B., Ramadhar T.R., Currie C.R., Clardy J. (2015). Variable genetic architectures produce virtually identical molecules in bacterial symbionts of fungus-growing ants. Proc. Natl. Acad. Sci. USA.

[99] Linneman J., Paulus D., Lim-Fong G., Lopanik N.B. (2014). Latitudinal variation of a defensive symbiosis in the *Bugula neritina* (Bryozoa) sibling species complex. PLoS ONE.

[100] Goodwin S., McPherson J.D., McCombie W.R. (2016). Coming of age: Ten years of next-generation sequencing technologies. Nat. Rev. Genet..

[101] Frank J.A., Pan Y., Tooming-Klunderud A., Eijsink V.G.H., McHardy A.C., Nederbragt A.J., Pope P.B. (2016). Improved metagenome assemblies and taxonomic binning using long-read circular consensus sequence data. Sci. Rep..

[102] Siegl A., Kamke J., Hochmuth T., Piel J., Richter M., Liang C., Dandekar T., Hentschel U. (2011). Single-cell genomics reveals the lifestyle of Poribacteria, a candidate phylum symbiotically associated with marine sponges. ISME J..

[103] Piel J. (2011). Approaches to capturing and designing biologically active small molecules produced by uncultured microbes. Annu. Rev. Microbiol..

[104] Lasken R.S. (2007). Single-cell genomic sequencing using Multiple Displacement Amplification. Curr. Opin. Microbiol..

[105] Blainey P.C., Quake S.R. (2011). Digital MDA for enumeration of total nucleic acid contamination. Nucleic Acids Res..

[106] Gawad C., Koh W., Quake S.R. (2016). Single-cell genome sequencing: Current state of the science. Nat. Rev. Genet..

[107] Pope P.B., Smith W., Denman S.E., Tringe S.G., Barry K., Hugenholtz P., McSweeney C.S., McHardy A.C., Morrison M. (2011). Isolation of Succinivibrionaceae implicated in low methane emissions from Tammar wallabies. Science.

[108] Wang S., Chng K.R., Wilm A., Zhao S., Yang K.-L., Nagarajan N., He J. (2014). Genomic characterization of three unique *Dehalococcoides* that respire on persistent polychlorinated biphenyls. Proc. Natl. Acad. Sci. USA.

[109] Cuív P.Ó., Smith W.J., Pottenger S., Burman S., Shanahan E.R., Morrison M. (2015). Isolation of genetically tractable most-wanted bacteria by metaparental mating. Sci. Rep..

[110] Tyson G.W., Lo I., Baker B.J., Allen E.E., Hugenholtz P., Banfield J.F. (2005). Genome-directed isolation of the key nitrogen fixer *Leptospirillum ferrodiazotrophum* sp. nov. from an acidophilic microbial community. Appl. Environ. Microbiol..

[111] Omsland A., Cockrell D.C., Howe D., Fischer E.R., Virtaneva K., Sturdevant D.E., Porcella S.F., Heinzen R.A. (2009). Host cell-free growth of the Q fever bacterium *Coxiella burnetii*. Proc. Natl. Acad. Sci. USA.

[112] Awan A.R., Shaw W.M., Ellis T. (2016). Biosynthesis of therapeutic natural products using synthetic biology. Adv. Drug Deliv. Rev..

[113] Owen J.G., Reddy B.V.B., Ternei M.A., Charlop-Powers Z., Calle P.Y., Kim J.H., Brady S.F. (2013). Mapping gene clusters within arrayed metagenomic libraries to expand the structural diversity of biomedically relevant natural products. Proc. Natl. Acad. Sci. USA.

[114] Lu Y.Y., Chen T., Fuhrman J.A., Sun F. (2016). COCACOLA: Binning metagenomic contigs using sequence Composition, read CoverAge, CO-alignment and paired-end read LinkAge. Bioinformatics.

[115] Chatterji S., Yamazaki I., Bai Z., Eisen J.A. (2008). CompostBin: A DNA composition-based algorithm for binning environmental shotgun reads. Research in Computational Molecular Biology.

[116] Alneberg J., Bjarnason B.S., de Bruijn I., Schirmer M., Quick J., Ijaz U.Z., Lahti L., Loman N.J., Andersson A.F., Quince C. (2014). Binning metagenomic contigs by coverage and composition. Nat. Methods.

[117] Sieber C.M.K., Probst A.J., Sharrar A., Thomas B.C., Hess M., Tringe S.G., Banfield J.F. (2017). Recovery of genomes from metagenomes via a dereplication, aggregation, and scoring strategy. bioRxiv.

[118] Imelfort M., Parks D., Woodcroft B.J., Dennis P., Hugenholtz P., Tyson G.W. (2014). GroopM: An automated tool for the recovery of population genomes from related metagenomes. PeerJ.

[119] Wu Y.-W., Simmons B.A., Singer S.W. (2016). MaxBin 2.0: An automated binning algorithm to recover genomes from multiple metagenomic datasets. Bioinformatics.

[120] Wang Y., Hu H., Li X. (2015). MBBC: An efficient approach for metagenomic binning based on clustering. BMC Bioinform..

[121] Kang D.D., Froula J., Egan R., Wang Z. (2015). MetaBAT, an efficient tool for accurately reconstructing single genomes from complex microbial communities. PeerJ.

[122] Strous M., Kraft B., Bisdorf R., Tegetmeyer H.E. (2012). The binning of metagenomic contigs for microbial physiology of mixed cultures. Front. Microbiol..

[123] Wang Y., Leung H.C.M., Yiu S.M., Chin F.Y.L. (2012). MetaCluster 5.0: A two-round binning approach for metagenomic data for low-abundance species in a noisy sample. Bioinformatics.

[124] Albertsen M., Hugenholtz P., Skarshewski A., Nielsen K.L., Tyson G.W., Nielsen P.H. (2013). Genome sequences of rare, uncultured bacteria obtained by differential coverage binning of multiple metagenomes. Nat. Biotechnol..

[125] Lin H.-H., Liao Y.-C. (2016). Accurate binning of metagenomic contigs via automated clustering sequences using information of genomic signatures and marker genes. Sci. Rep..

[126] Mohammed M.H., Ghosh T.S., Singh N.K., Mande S.S. (2011). SPHINX—An algorithm for taxonomic binning of metagenomic sequences. Bioinformatics.

[127] Kelley D.R., Salzberg S.L. (2010). Clustering metagenomic sequences with interpolated Markov models. BMC Bioinform..

[128] Ultsch A., Mörchen F. (2005). ESOM-Maps: Tools for Clustering, Visualization, and Classification with Emergent SOM.

[129] Dick G.J., Andersson A.F., Baker B.J., Simmons S.L., Thomas B.C., Yelton A.P., Banfield J.F. (2009). Community-wide analysis of microbial genome sequence signatures. Genome Biol..

[130] Laczny C.C., Sternal T., Plugaru V., Gawron P., Atashpendar A., Margossian H.H., Coronado S., van der Maaten L., Vlassis N., Wilmes P. (2015). VizBin—An application for reference-independent visualization and human-augmented binning of metagenomic data. Microbiome.

[131] Saeed I., Tang S.-L., Halgamuge S.K. (2012). Unsupervised discovery of microbial population structure within metagenomes using nucleotide base composition. Nucleic Acids Res..

[132] Nielsen H.B., Almeida M., Juncker A.S., Rasmussen S., Li J., Sunagawa S., Plichta D.R., Gautier L., Pedersen A.G., Le Chatelier E. (2014). MetaHIT Consortium Identification and assembly of genomes and genetic elements in complex metagenomic samples without using reference genomes. Nat. Biotechnol..

[133] Wang Y., Leung H., Yiu S., Chin F. (2014). MetaCluster-TA: Taxonomic annotation for metagenomic data based on assembly-assisted binning. BMC Genom..

[134] Sedlar K., Kupkova K., Provaznik I. (2017). Bioinformatics strategies for taxonomy independent binning and visualization of sequences in shotgun metagenomics. Comput. Struct. Biotechnol. J..

[135] Cheng T.Y., Sueoka N. (1963). Heterogeneity of DNA in density and base composition. Science.

[136] Teeling H., Meyerdierks A., Bauer M., Amann R., Glöckner F.O. (2004). Application of tetranucleotide frequencies for the assignment of genomic fragments. Environ. Microbiol..

[137] Laczny C.C., Pinel N., Vlassis N., Wilmes P. (2014). Alignment-free visualization of metagenomic data by nonlinear dimension reduction. Sci. Rep..

[138] Medini D., Donati C., Tettelin H., Masignani V., Rappuoli R. (2005). The microbial pan-genome. Curr. Opin. Genet. Dev..

[139] Tettelin H., Riley D., Cattuto C., Medini D. (2008). Comparative genomics: The bacterial pan-genome. Curr. Opin. Microbiol..

[140] Miller I.J., Weyna T.R., Fong S.S., Lim-Fong G.E., Kwan J.C. (2016). Single sample resolution of rare microbial dark matter in a marine invertebrate metagenome. Sci. Rep..

[141] Gurevich A., Saveliev V., Vyahhi N., Tesler G. (2013). QUAST: Quality assessment tool for genome assemblies. Bioinformatics.

[142] Mikheenko A., Saveliev V., Gurevich A. (2016). MetaQUAST: Evaluation of metagenome assemblies. Bioinformatics.

[143] Hunt M., Kikuchi T., Sanders M., Newbold C., Berriman M., Otto T.D. (2013). REAPR: A universal tool for genome assembly evaluation. Genome Biol..

[144] Walker B.J., Abeel T., Shea T., Priest M., Abouelliel A., Sakthikumar S., Cuomo C.A., Zeng Q., Wortman J., Young S.K. (2014). Pilon: An integrated tool for comprehensive microbial variant detection and genome assembly improvement. PLoS ONE.

[145] Thomas T., Gilbert J., Meyer F. (2012). Metagenomics—A guide from sampling to data analysis. Microb. Inform. Exp..

[146] Sangwan N., Xia F., Gilbert J.A. (2016). Recovering complete and draft population genomes from metagenome datasets. Microbiome.

[147] Ashton P.M., Nair S., Dallman T., Rubino S., Rabsch W., Mwaigwisya S., Wain J., O’Grady J. (2015). MinION nanopore sequencing identifies the position and structure of a bacterial antibiotic resistance island. Nat. Biotechnol..

[148] Sharon I., Kertesz M., Hug L.A., Pushkarev D., Blauwkamp T.A., Castelle C.J., Amirebrahimi M., Thomas B.C., Burstein D., Tringe S.G. (2015). Accurate, multi-kb reads resolve complex populations and detect rare microorganisms. Genome Res..

[149] Burton J.N., Liachko I., Dunham M.J., Shendure J. (2014). Species-level deconvolution of metagenome assemblies with Hi-C-based contact probability maps. G3.

[150] Marbouty M., Baudry L., Cournac A., Koszul R. (2017). Scaffolding bacterial genomes and probing host-virus interactions in gut microbiome by proximity ligation (chromosome capture) assay. Sci. Adv..

[151] Beitel C.W., Froenicke L., Lang J.M., Korf I.F., Michelmore R.W., Eisen J.A., Darling A.E. (2014). Strain- and plasmid-level deconvolution of a synthetic metagenome by sequencing proximity ligation products. PeerJ.

[152] Flot J.-F., Marie-Nelly H., Koszul R. (2015). Contact genomics: Scaffolding and phasing (meta)genomes using chromosome 3D physical signatures. FEBS Lett..

[153] Liu M., Darling A. (2015). Metagenomic Chromosome Conformation Capture (3C): Techniques, applications, and challenges. F1000Research.

[154] Marbouty M., Cournac A., Flot J.-F., Marie-Nelly H., Mozziconacci J., Koszul R. (2014). Metagenomic chromosome conformation capture (meta3C) unveils the diversity of chromosome organization in microorganisms. eLife.

[155] Staunton J., Weissman K.J. (2001). Polyketide biosynthesis: A millennium review. Nat. Prod. Rep..

[156] Kamra P., Gokhale R.S., Mohanty D. (2005). SEARCHGTr: A program for analysis of glycosyltransferases involved in glycosylation of secondary metabolites. Nucleic Acids Res..

[157] Hertweck C. (2009). The biosynthetic logic of polyketide diversity. Angew. Chem. Int. Ed. Engl..

[158] Strieker M., Tanović A., Marahiel M.A. (2010). Nonribosomal peptide synthetases: Structures and dynamics. Curr. Opin. Struct. Biol..

[159] Yadav G., Gokhale R.S., Mohanty D. (2003). Computational approach for prediction of domain organization and substrate specificity of modular polyketide synthases. J. Mol. Biol..

[160] Irschik H., Kopp M., Weissman K.J., Buntin K., Piel J., Müller R. (2010). Analysis of the sorangicin gene cluster reinforces the utility of a combined phylogenetic/retrobiosynthetic analysis for deciphering natural product assembly by *trans*-AT PKS. Chembiochem.

[161] Jensen K., Niederkrüger H., Zimmermann K., Vagstad A.L., Moldenhauer J., Brendel N., Frank S., Pöplau P., Kohlhaas C., Townsend C.A. (2012). Polyketide proofreading by an acyltransferase-like enzyme. Chem. Biol..

[162] Challis G.L., Ravel J., Townsend C.A. (2000). Predictive, structure-based model of amino acid recognition by nonribosomal peptide synthetase adenylation domains. Chem. Biol..

[163] Piel J. (2010). Biosynthesis of polyketides by *trans*-AT polyketide synthases. Nat. Prod. Rep..

[164] Sharp K.H., Davidson S.K., Haygood M.G. (2007). Localization of “*Candidatus* Endobugula sertula” and the bryostatins throughout the life cycle of the bryozoan *Bugula neritina*. ISME J..

[165] Udwary D.W., Zeigler L., Asolkar R.N., Singan V., Lapidus A., Fenical W., Jensen P.R., Moore B.S. (2007). Genome sequencing reveals complex secondary metabolome in the marine actinomycete *Salinispora tropica*. Proc. Natl. Acad. Sci. USA.

[166] Lane A.L., Moore B.S. (2011). A sea of biosynthesis: Marine natural products meet the molecular age. Nat. Prod. Rep..

[167] Matsumori N., Kaneno D., Murata M., Nakamura H., Tachibana K. (1999). Stereochemical determination of acyclic structures based on carbon-proton spin-coupling constants. A method of configuration analysis for natural products. J. Org. Chem..

[168] Lei H., Yan J., Yu J., Liu Y., Wang Z., Xu Z., Ye T. (2014). Total synthesis and stereochemical reassignment of mandelalide A. Angew. Chem. Int. Ed. Engl..

[169] Huang W., Li L., Myers J.R., Marth G.T. (2012). ART: A next-generation sequencing read simulator. Bioinformatics.

[170] Ono Y., Asai K., Hamada M. (2013). PBSIM: PacBio reads simulator—Toward accurate genome assembly. Bioinformatics.

[171] Mirebrahim H., Close T.J., Lonardi S. (2015). *De novo* meta-assembly of ultra-deep sequencing data. Bioinformatics.

[172] Lonardi S., Mirebrahim H., Wanamaker S., Alpert M., Ciardo G., Duma D., Close T.J. (2015). When less is more: “Slicing” sequencing data improves read decoding accuracy and *de novo* assembly quality. Bioinformatics.

[173] Reen F.J., Gutiérrez-Barranquero J.A., Dobson A.D.W., Adams C., O’Gara F. (2015). Emerging concepts promising new horizons for marine biodiscovery and synthetic biology. Mar. Drugs.

[174] Kennedy J., Flemer B., Jackson S.A., Lejon D.P.H., Morrissey J.P., O’Gara F., Dobson A.D.W. (2010). Marine metagenomics: New tools for the study and exploitation of marine microbial metabolism. Mar. Drugs.

[175] Wilson M.C., Piel J. (2013). Metagenomic approaches for exploiting uncultivated bacteria as a resource for novel biosynthetic enzymology. Chem. Biol..

[176] Suenaga H. (2012). Targeted metagenomics: A high-resolution metagenomics approach for specific gene clusters in complex microbial communities. Environ. Microbiol..

[177] Wilson M.C., Mori T., Rückert C., Uria A.R., Helf M.J., Takada K., Gernert C., Steffens U.A.E., Heycke N., Schmitt S. (2014). An environmental bacterial taxon with a large and distinct metabolic repertoire. Nature.

[178] Baker M. (2012). *De novo* genome assembly: What every biologist should know. Nat. Methods.

[179] Sczyrba A., Hofmann P., Belmann P., Koslicki D. (2017). Critical Assessment of Metagenome Interpretation—A benchmark of computational metagenomics software. bioRxiv.

[180] Medema M.H., Kottmann R., Yilmaz P., Cummings M., Biggins J.B., Blin K., de Bruijn I., Chooi Y.H., Claesen J., Coates R.C. (2015). Minimum Information about a Biosynthetic Gene cluster. Nat. Chem. Biol..

[181] Garza D.R., Dutilh B.E. (2015). From cultured to uncultured genome sequences: Metagenomics and modeling microbial ecosystems. Cell. Mol. Life Sci..

[182] Klitgord N., Segrè D. (2010). Environments that induce synthetic microbial ecosystems. PLoS Comput. Biol..

