# Supplementary material for: Interpreting Microbial Biosynthesis in the Genomic Age: Biological and Practical Considerations
Source: Mar Drugs. 2017 Jun 6;15(6):165. doi: 10.3390/md15060165 (PMC5484115; doi:10.3390/md15060165)
Supplement: Supplementary file 1 [file marinedrugs-15-00165-s001.pdf]

Supplementary Information

Table of Contents **Page**

Supplemental Methods. Simulation of *Salinispora tropica* sequencing and salinilactam (*slm*) biosynthetic gene cluster analysis S1

Table S1. Quantitative data on *Salinispora tropica* genome statistics based on simulated sequencing parameters. S2

Figure S1. Fragmentation (no. contigs) and percent recovery of the salinilactm (*slm*) biosynthetic gene cluster based on expanded set of simulated Illumina and PacBio sequencing parameters. S3

Figure S2. (a) Nx (where x is 0 - 100% of the assembly length) and (b) cumulative length plots for assemblies using a read length of 50 bp, insert size of 275 bp and ranges of sequencing depth (1 - 1000×). S4

Supplemental References. S5

Supplemental Methods

Simulation of Salinispora tropica *sequencing and salinilactam* (slm*) biosynthetic gene cluster analysis*

Below are the steps to simulate Illumina HiSeq 2500 and PacBio RS sequencing of the *Salinispora tropica* genome. The example shown here is for a simulation with and without PacBio sequencing using PBSIM (v1.0.3) [1] with a 30✕ depth of sequencing, CLR mode; and an Illumina sequencing (v2.5.1) [2] read length 125, fragment size 275 (stdev=90), and 100✕ depth of sequencing. The simulated sequencing datasets were assembled with SPAdes (v3.9.0) [3]

1. Simulate reads
   - Illumina only (insert size 275 bp):
     i. art_illumina -p -ss HS25 -l 125 -f 100 -o len125_cov100 -m 275 -s 90 -i GCA_000016425.1__ASM1642v1_genomic.fna
   - With PacBio:
     i. pbsim --data-type CLR --depth 30 --model_qc model_qc_clr GCA_000016425.1_ASM1642v1_genomic.fna
2. Assemble with SPAdes
   - Illumina only:
     i. spades.py -1 len125_cov100_reads1.fq.gz -2 len125_cov100_reads2.fq.gz -t 16 -m 16 -o S_tropica_len100_cov100_spades_asm
   - With PacBio:
     i. spades.py -1 len125_cov100_simulated_reads1.fq.gz -2 len125_cov100_simulated_reads2.fq.gz --pacbio sd_0001.fastq -t 16 -m 16 -o Salinospora_tropica_len125_cov100_pb30X_spades_asm
3. Align *de novo* contigs (from Illumina only run) to reference genome and calculate fragmentation based on alignment using Python script
   - calculate_pathway_fragmentation.py -a len125_cov100_scaffolds.fasta -r GCA_000016425.1_ASM1642v1_genomic.fna -p s_tropica_cluster_coordinates.tab

Source code for calculate_pathway_fragmentation.py and s_tropica_cluster_coordinates.tab adapted from ORF coordinates in Table 2 of Udwary et al. [4] avaliable at https://github.com/ijmiller2/salinilactam_BGC_analysis.

Table S1. Quantitative data on *Salinispora tropica* genome assembly statistics based on simulated sequencing parameters.

parameters	length (Mbp)	no. contigs	N50 (Kbp)	largest seq (Kbp)	Ns (bp)	%GC
len125_cov1	1.59	4747	0.3	1.9	450	69.43
len125_cov10	5.14	195	117.4	381.5	265	69.50
len125_cov100	5.14	161	217.8	441.9	759	69.50
len125_cov1000	5.14	163	217.9	430.6	666	69.50
len100_cov1	0.96	3038	0.3	2.0	1032	69.36
len100_cov10	5.14	206	141.8	427.8	652	69.50
len100_cov100	5.14	167	217.8	427.4	623	69.50
len100_cov1000	5.14	166	217.9	427.6	720	69.50
len50_cov1	0.01	65	0.2	1.2	40	67.49
len50_cov10	5.12	370	75.7	297.4	4861	69.45
len50_cov100	5.13	337	202.5	350.3	249	69.51
len50_cov1000	5.13	317	202.5	350.4	233	69.51
len125_cov1_frag1000	1.81	4298	0.4	4.1	346090	56.28
len125_cov10_frag1000	5.14	179	254.7	427.5	2333	69.48
len125_cov100_frag1000	5.15	149	364.5	596.5	1335	69.49
len125_cov1000_frag1000	5.15	141	364.5	596.5	1252	69.49
len125_cov100_pb10X	5.15	77	201.2	638.9	90	69.50
len125_cov100_pb20X	5.17	41	355.3	921.8	100	69.49
len125_cov100_pb30X	5.17	36	588.6	1110.5	100	69.49
len125_cov100_pb50X	5.18	39	936.2	983.1	100	69.47

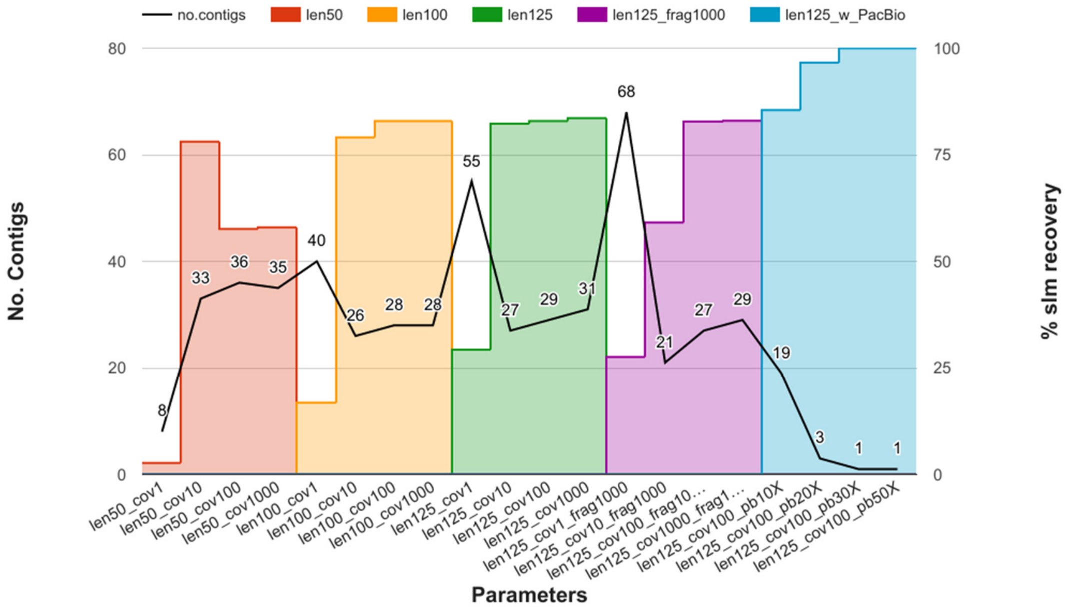

Figure S1. Fragmentation (no. contigs) and percent recovery of the salinilactam (*slm*) biosynthetic gene cluster based on expanded set of simulated Illumina and PacBio sequencing parameters.

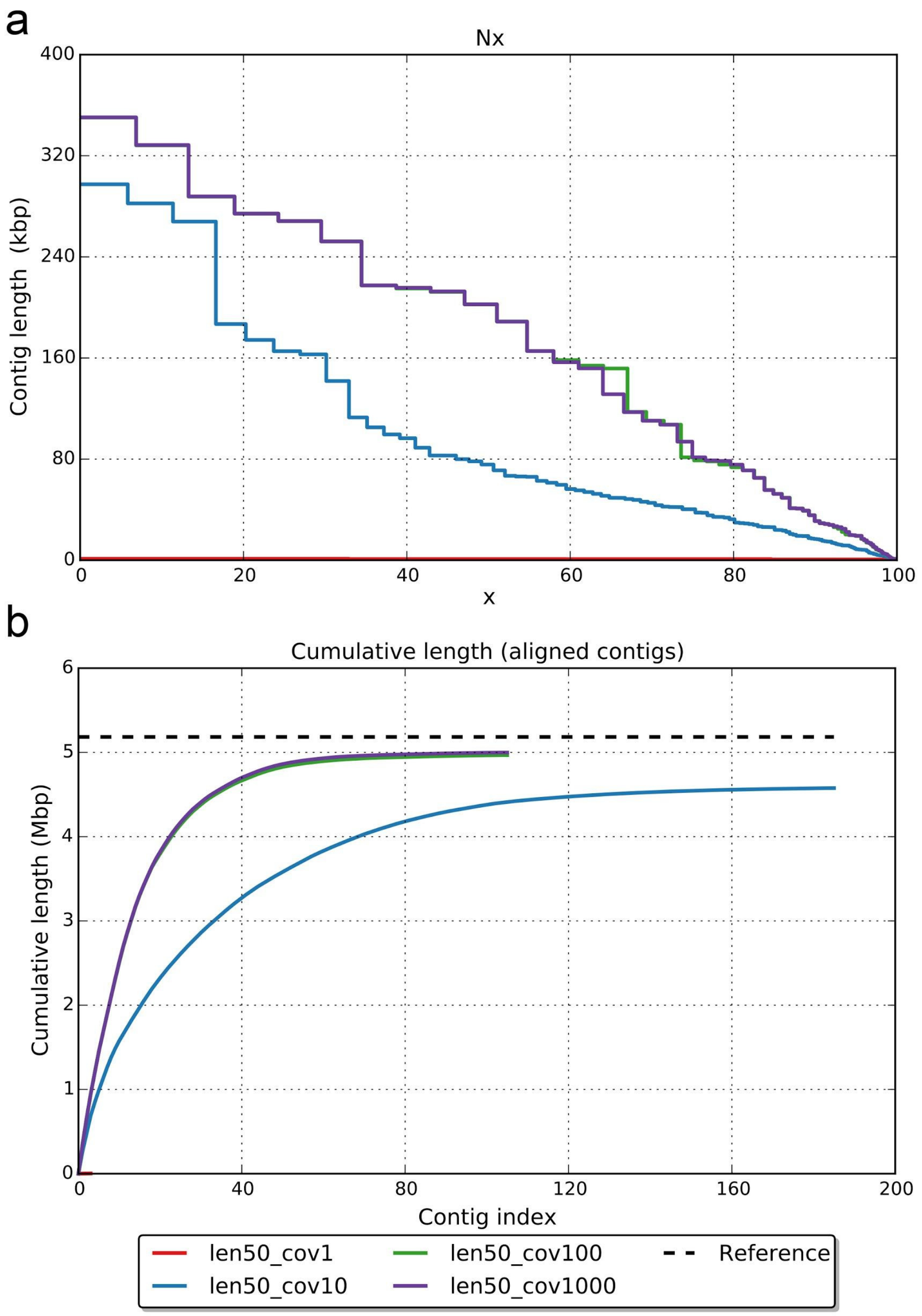

Figure S2. (a) Nx (where x is 0 - 100% of the assembly length) and (b) cumulative length plots for assemblies using a read length of 50 bp, insert size of 275 bp and ranges of sequencing depth (1 - 1000×) produced using QUAST (v4.1) [5].

Supplemental References

1. Ono, Y.; Asai, K.; Hamada, M. PBSIM: PacBio reads simulator—toward accurate genome assembly. *Bioinformatics* **2013**, *29*, 119–121.
2. Huang, W.; Li, L.; Myers, J. R.; Marth, G. T. ART: A next-generation sequencing read simulator. *Bioinformatics* **2012**, *28*, 593–594.
3. Bankevich, A.; Nurk, S.; Antipov, D.; Gurevich, A. A.; Dvorkin, M.; Kulikov, A. S.; Lesin, V. M.; Nikolenko, S. I.; Pham, S.; Prjibelski, A. D.; Pyshkin, A. V.; Sirotkin, A. V.; Vyahhi, N.; Tesler, G.; Alekseyev, M. A.; Pevzner, P. A. SPAdes: A new genome assembly algorithm and its applications to single-cell sequencing. *J. Comput. Biol.* **2012**, 19, 455–477.
4. Udwary, D. W.; Zeigler, L.; Asolkar, R. N.; Singan, V.; Lapidus, A.; Fenical, W.; Jensen, P. R.; Moore, B. S. Genome sequencing reveals complex secondary metabolome in the marine actinomycete *Salinispora tropica*. Proc. Natl. Acad. Sci. U. S. A. 2007, 104, 10376–10381.
5. Gurevich, A.; Saveliev, V.; Vyahhi, N.; Tesler, G. QUAST: Quality assessment tool for genome assemblies. *Bioinformatics* 2013, 29, 1072–1075.

© 2017 by the authors. Submitted for possible open access publication under the terms and conditions of the Creative Commons Attribution (CC BY) license (http://creativecommons.org/licenses/by/4.0/).
